# The YIN and YANG of lipoproteins in developing and preventing infectious arthritis by *Staphylococcus aureus*

**DOI:** 10.1371/journal.ppat.1007877

**Published:** 2019-06-21

**Authors:** Majd Mohammad, Minh-Thu Nguyen, Cecilia Engdahl, Manli Na, Anders Jarneborn, Zhicheng Hu, Anna Karlsson, Rille Pullerits, Abukar Ali, Friedrich Götz, Tao Jin

**Affiliations:** 1 Department of Rheumatology and Inflammation Research, Institute of Medicine, Sahlgrenska Academy, University of Gothenburg, Gothenburg, Sweden; 2 Department of Microbial Genetics, University of Tübingen, Tübingen, Germany; 3 Department of Rheumatology, Sahlgrenska University Hospital, Gothenburg, Sweden; 4 Department of Microbiology and Immunology, The Affiliated Hospital of Guizhou Medical University, Guiyang, China; 5 Department of Clinical Immunology and Transfusion Medicine, Sahlgrenska University Hospital, Gothenburg, Sweden; University of Nebraska Medical Center, UNITED STATES

## Abstract

Rapid bone destruction often leads to permanent joint dysfunction in patients with septic arthritis, which is mainly caused by *Staphylococcus aureus* (*S*. *aureus*). Staphylococcal cell wall components are known to induce joint inflammation and bone destruction. Here, we show that a single intra-articular injection of *S*. *aureus* lipoproteins (Lpps) into mouse knee joints induced chronic destructive macroscopic arthritis through TLR2. Arthritis was characterized by rapid infiltration of neutrophils and monocytes. The arthritogenic effect was mediated mainly by macrophages/monocytes and partially via TNF-α but not by neutrophils. Surprisingly, a *S*. *aureus* mutant lacking Lpp diacylglyceryl transferase (*lgt*) caused more severe joint inflammation, which coincided with higher bacterial loads of the *lgt* mutant in local joints than those of its parental strain. Coinjection of pathogenic *S*. *aureus* LS-1 with staphylococcal Lpps into mouse knee joints caused improved bacterial elimination and diminished bone erosion. The protective effect of the Lpps was mediated by their lipid moiety and was fully dependent on TLR2 and neutrophils. The blocking of CXCR2 on neutrophils resulted in total abrogation of the protective effect of the Lpps. Our data demonstrate that *S*. *aureus* Lpps elicit innate immune responses, resulting in a double-edged effect. On the one hand, staphylococcal Lpps boost septic arthritis. On the other hand, Lpps act as adjuvants and activate innate immunity, which could be useful for combating infections with multiple drug-resistant strains.

## Author summary

Rapid bone destruction often leads to permanent joint dysfunction in septic arthritis, which is mainly caused by *S*. *aureus*. Despite advances in the use of antibiotics, permanent reductions in joint function occur in up to 50% of patients, who may also need joint replacement surgery. Additional challenge is posed by increasing antibiotic resistance of *S*. *aureus*, causing significant clinical and economic consequences. Although the outcome is poor, the current treatments for septic arthritis remain unchanged since many decades. It is largely unknown which bacterial factors cause aggressive joint damage. Here, we show that a single intra-articular injection of *S*. *aureus* lipoproteins (Lpps) into mouse knee joints induced chronic destructive macroscopic arthritis, and the monocytes/macrophages were the main culprit. However, coinjection of pathogenic *S*. *aureus* with Lpps into mouse knee joints attenuated the disease. The protective effect of Lpps was mediated by their lipid moiety, TLR2 on the host cells, neutrophil chemokine release, and consequent neutrophil recruitment. Our finding might be used as a novel concept in the treatment of multidrug-resistant bacterial infections.

## Introduction

Despite colonizing more than half of the human population at some stage during their life [1], *Staphylococcus aureus* (*S*. *aureus*) is a highly pathogenic microorganism responsible for a broad range of infections in humans [2]. Septic arthritis, considered to be one of the most aggressive joint diseases, is most commonly caused by *S*. *aureus* [3]. In a mouse model, *S*. *aureus* induced severe bone destruction 5 days postinfection [4]. In patients, even after initiating immediate treatment, the joint damage caused by septic arthritis is often irreversible [5], leading to permanent joint dysfunction in up to half of the patients [6]. Furthermore, the emergence of methicillin-resistant *S*. *aureus* (MRSA) has severely reduced the available treatment options [2, 7]. To limit the immune response and reduce the risk of permanent joint destruction, a combination treatment of antibiotics with immunomodulatory therapy has been proposed [8, 9]. However, there are potential dangers associated with such combination therapies as long as the challenge of antibiotic resistance remains [10, 11]. Therefore, the identification of the bacterial components responsible for joint inflammation and destruction is key for the development of new therapies. Antibiotic-killed *S*. *aureus* is known to induce destructive arthritis, and the bacterial cell wall components are the culprits in this context [12]. Among the *S*. *aureus* cell wall components, lipoproteins (Lpps) are Toll-like receptor 2 (TLR2) agonists and the main immune stimulators, while lipoteichoic acids are much less important [13–15]. Lipidation of Lpps is known to be crucial for virulence in murine *S*. *aureus* systemic infection [16]. Depending on the degree of acylation in the lipid moiety of the Lpps, different TLR2 receptor combinations are activated: triacylated Lpps are agonists of TLR2/TLR1 heterodimers, while diacylated Lpps are agonists of TLR2/TLR6 heterodimers [17, 18]. Staphylococcal species differ in the length of the fatty acid in the N-acyl group of the lipid moiety, which has drastic effects on innate and adaptive immune stimulation [19].

In the present study, we hypothesized that staphylococcal Lpps are the main inducer of synovitis and joint destruction in *S*. *aureus*-induced septic arthritis. Indeed, a single intra-articular injection of *S*. *aureus* Lpps induced macroscopic, chronic and destructive arthritis, which was mediated by monocytes/macrophages. However, the *Δlgt* strain, an Lpp diacylglyceryl transferase (*lgt*) deletion mutant, caused more severe joint inflammation than its parental strain. This increased severity in joint inflammation was due to the better survival of the *Δlgt* strain, suggesting that a lack of Lpps induces immune evasion. Importantly, coinjection of *S*. *aureus* with staphylococcal Lpps in mouse knee joints resulted in radical elimination of bacteria and diminished bone erosion. This protective effect was mediated by the lipid moiety of the Lpps and was fully dependent on TLR2 and the recruitment of neutrophils.

## Results

### Staphylococcal lipoproteins induce macroscopic, chronic and destructive arthritis

**Fig 1:**
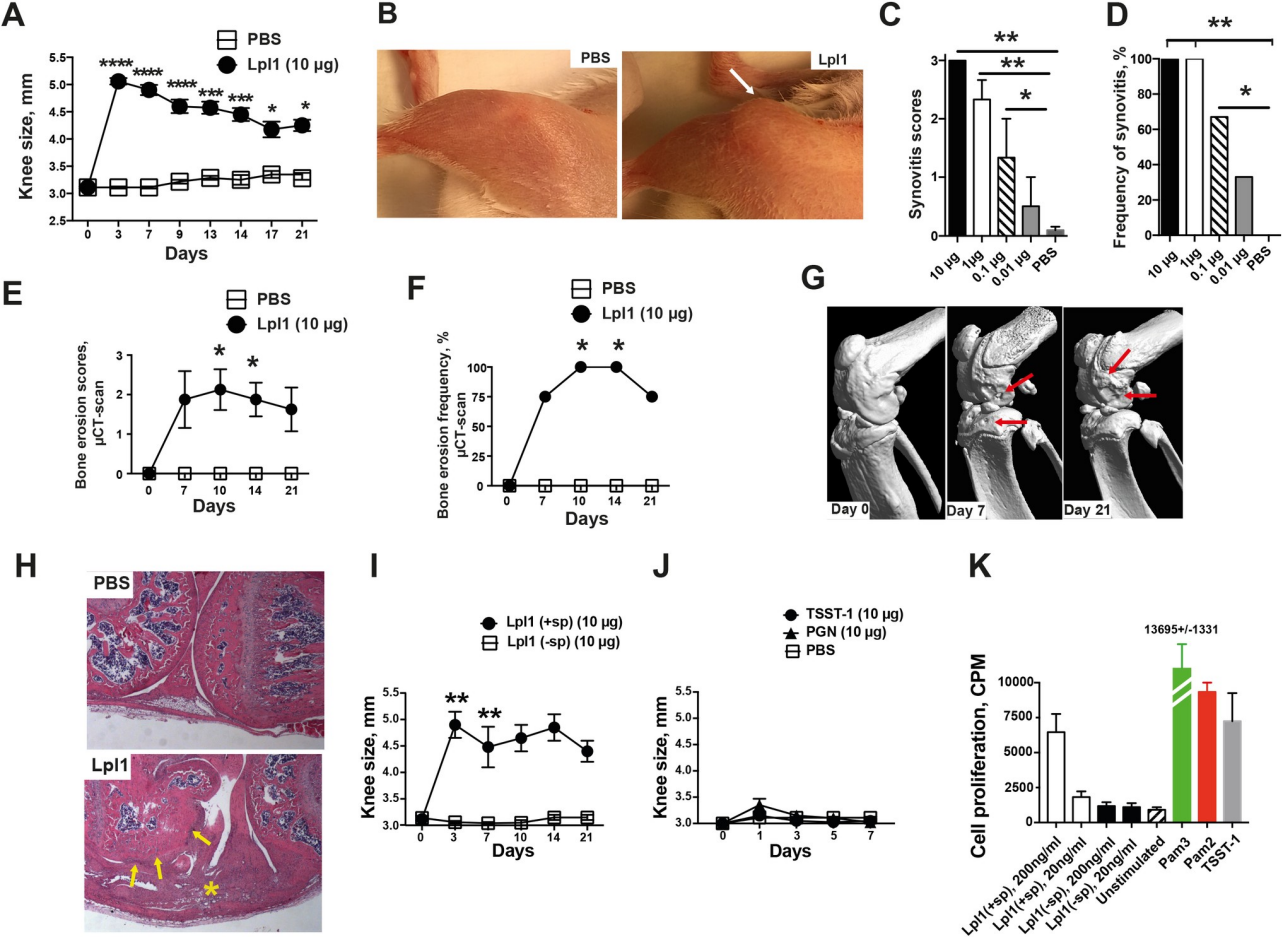
Staphylococcal lipoproteins induce destructive long-lasting arthritis. **(A)** Measurement of knee swelling in millimeters (mm) of NMRI mice (n = 4-12/group) up to 21 days after intra-articular (i.a.) knee joint injection with 20 μl of phosphate-buffered saline (PBS) or purified staphylococcal lipoprotein, denoted as Lpl1(+sp) (10 μg/knee). **(B)** Representative images of knee joints i.a. injected with PBS (left panel) or Lpl1(+sp) (right panel) on day 3 after i.a. injection. The severity **(C)** and frequency **(D)** of synovitis in NMRI mice on day 3 after i.a. injection with various concentrations (0.01–10 μg/knee) of Lpl1(+sp) (n = 3/group) or PBS (n = 11). The severity **(E)** and cumulative frequency **(F)** of bone erosion evaluated with a microcomputed tomography (μCT) scan overtime in NMRI mouse knee joints following i.a. injection of Lpl1(+sp) (10 μg/knee). **(G)** Representative μCT images of NMRI mouse knee joints before i.a. injection (day 0) and after (day 7 and 21) i.a. injection of Lpl1(+sp) (10 μg/knee). Arrows indicate bone erosion. **(H)** Representative photomicrographs of knee joints of NMRI mice receiving Lpl1(+sp) (10 μg/knee) or PBS on day 14, stained with hematoxylin and eosin. Arrows indicate bone erosion, and * indicates inflamed synovium. Measurement of knee swelling (mm) in NMRI mice (n = 2-5/group) following i.a. knee injection of **(I)** Lpl1(+sp) or unlipidated Lpl1 protein, denoted as Lpl1(-sp) (10 μg/knee), or **(J)** Toxic shock syndrome toxin-1 (TSST-1, 10 μg/knee), peptidoglycan (PGN, 10 μg/knee), or PBS (n = 4-10/group). **(K)** Proliferative responses of splenocytes to Lpl1(+sp), Lpl1(-sp) (20–200 ng/mL) and lipopeptides (Pam2CSK4 and Pam3CSK4, 40 ng/mL) (n = 5/group). TSST-1 (100 ng/mL) and culture medium was used as positive and negative controls, respectively. Statistical evaluations were performed using the Mann–Whitney U test, with data expressed as the mean ± standard error of the mean (A, C, E, I, J and K), or Fisher’s exact test (D and F). *P < 0.05; **P < 0.01; ***P < 0.001; ****P < 0.0001.

We injected the purified staphylococcal Lpp Lpl1 intra-articularly (i.a.) into mouse knee joints. Lpl1 is a model Lpp derived from the νSaα-specific lipoprotein-like cluster (*lpl*) that exists in highly pathogenic and epidemic *S*. *aureus* strains [20]. One single injection of Lpl1 at a dose of 10 μg/knee caused macroscopic joint inflammation after 24 hours, and the inflammation lasted for at least 21 days (Fig 1A and 1B). The arthritogenic effect of Lpl1 was dose-dependent—even a 100-fold lower dose (0.1 μg/knee) induced synovitis (Fig 1C and 1D). Importantly, severe bone erosions were observed on day 7, and all Lpl1-injected joints had erosions on day 10, as verified by microcomputed tomography (μCT) scans (Fig 1E–1G). Histologically, the highly inflamed synovium, pannus formation, and severe bone destruction (Fig 1H) exhibited characteristics of the typical histopathological picture of *S*. *aureus* septic arthritis [21]. The minimal bone destruction-inducing dose of Lpl1 was much higher (10 μg/knee) than the inflammation-inducing dose (0.1 μg/knee). To further understand whether the lipid- or protein-moiety of Lpl1 was responsible for the arthritogenic effect, Lpp lacking the lipid moiety Lpl1(-sp) was compared to the intact Lpp, Lpl1(+sp). Lpl1(-sp) completely lacked the capacity to induce arthritis (Fig 1I), suggesting that the lipid moiety of staphylococcal Lpps is fully responsible for their arthritogenic properties. Indeed, *in vitro* splenocyte proliferation was induced by both Lpl1(+sp) and Pam3CSK4 (a synthetic lipopeptide mimicking the N-terminal lipid portion of Lpps) but not by Lpl1(-sp) (Fig 1K). To compare the arthritogenic capacity of Lpps with other *S*. *aureus* components, we injected mice i.a. with the staphylococcal superantigen toxic shock syndrome toxin-1 (TSST-1) and with the peptidoglycan (PGN) purified from a mutant strain lacking Lpp diacylglyceryl transferase [15]. Only very mild and transient knee joint swelling was observed on day 1, and the swelling disappeared by day 3 in the knee joints injected with 10 μg of PGN (Fig 1J). Interestingly, heat-treated Lpl1 and Pam3CSK4 preserved their arthritogenic capacity (S1 Fig), strongly suggesting that the Lpl1 is heat-insensitive.

### Neutrophils and monocytes are rapidly recruited to the joints after intra-articular injection of lipoproteins

**Fig 2:**
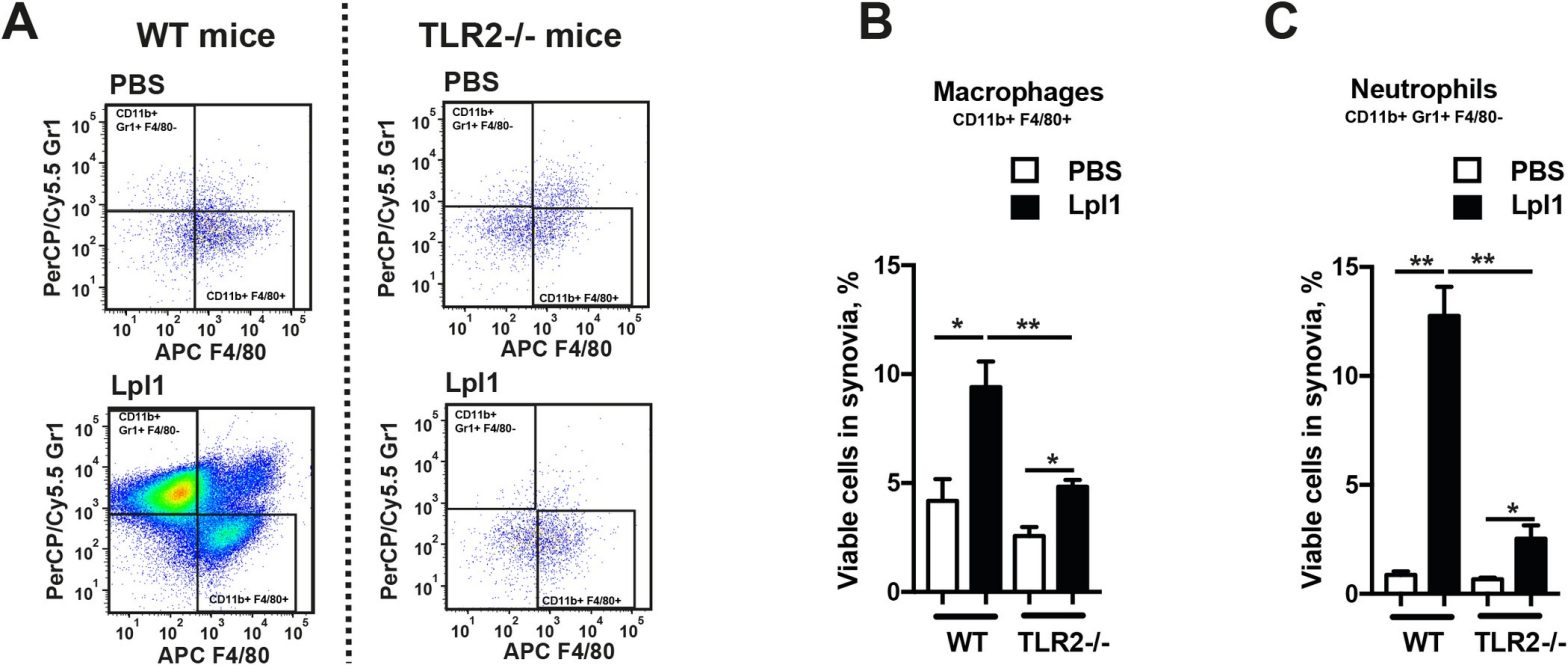
Neutrophils and monocytes migrate to the knee joints injected with staphylococcal lipoproteins. **(A)** Representative images of fluorescence-activated cell sorting (FACS) analysis demonstrating the migration of monocytes/macrophages (CD11b+F4/80+) and neutrophils (CD11b+Gr1+F4/80-) to synovial tissues following intra-articular (i.a.) injection of PBS or purified *S*. *aureus* lipoprotein, denoted as Lpl1 (5 μg/knee) in C57BL/6 wild-type (WT, left panel) and TLR2-deficient (TLR2-/-, right panel) mice. Frequencies of monocytes/macrophages **(B)** and neutrophils **(C)** in the knee joints of C57BL/6 WT and TLR2-/- mice (n = 5/group) on day 1 after i.a. injected with 20 μl of PBS or Lpl1 (5 μg/knee, n = 5/group). Statistical evaluations were performed using the Mann–Whitney U test, with data expressed as the mean ± standard error of the mean. *P < 0.05; **P < 0.01.

To understand the cellular mechanism behind Lpp-induced arthritis, we further analyzed the immune cells present in the local synovium using flow cytometry one day after Lpl1 injection. Synovial tissues from Lpl1-injected knee joints of wild-type mice demonstrated higher numbers and frequencies of CD11b+F4/80+ cells (monocytes/macrophages) and CD11b+Gr1+F4/80- cells (neutrophils) than those of phosphate-buffered saline (PBS)-injected knee joints. Significantly decreased numbers of infiltrating monocytes/macrophages and neutrophils were observed in TLR2-deficient (TLR2^-/-^) mice (Fig 2A–2C). No difference with regard to B- and T-cells was observed. These results suggest that TLR2 is highly important for neutrophil and monocyte recruitment into synovial tissue following Lpl1 injection.

### Staphylococcal lipoprotein-induced arthritis is mediated by monocytes/macrophages through TLR2

**Fig 3:**
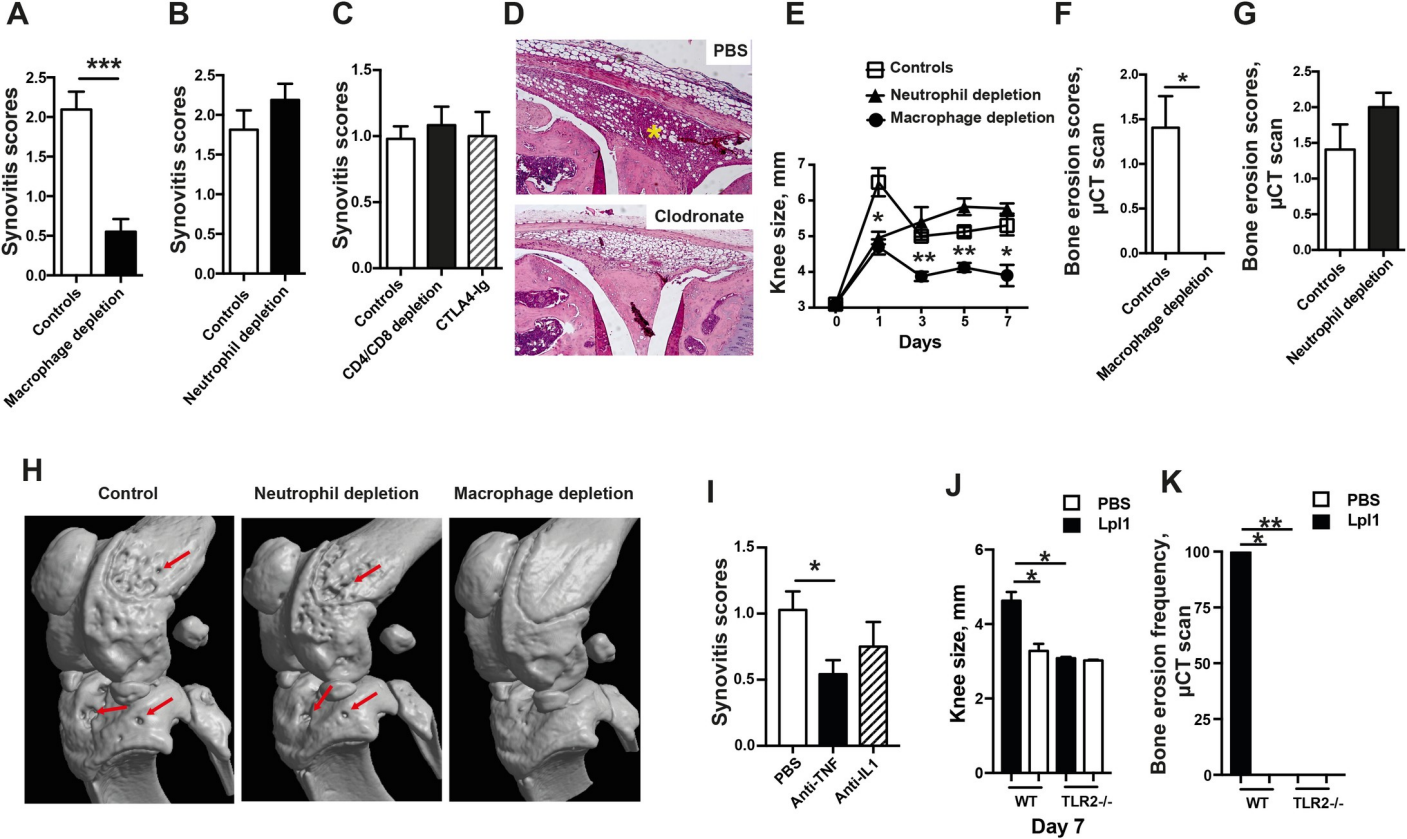
Staphylococcal lipoprotein-induced arthritis is dependent on monocytes/macrophages, TNF, and TLR2. Synovitis severity induced by intra-articular (i.a.) knee injection of 20 μl of purified *S*. *aureus* lipoprotein, denoted as Lpl1 (0.33 μg/knee) on day 3 in NMRI mice **(A)** depleted of monocytes/macrophages using clodronate liposomes (n = 8-10/group); **(B)** depleted of neutrophils using anti-Ly6G antibody (n = 8/group); **(C)** depleted of T-cells using anti-CD4 and anti-CD8 antibodies simultaneously or blocking T-cell activation using abatacept, a fusion protein of CTLA4-Ig (n = 6-12/group). **(D)** Representative photomicrographs of Lpl1-injected knee joints from NMRI mice depleted of monocytes/macrophages stained with hematoxylin and eosin. * indicates inflamed synovium. **(E)** Measurement of knee swelling (mm) of NMRI mice depleted of monocytes/macrophages or neutrophils after i.a. knee injection with 20 μl of Lpl1 (10 μg/knee). Bone erosion induced by i.a. knee joint injection of 20 μl of Lpl1 (10 μg/knee) in NMRI mice **(F)** depleted of monocytes/macrophages using clodronate liposomes (n = 4-8/group) or **(G)** depleted of neutrophils using anti-Ly6G antibody (n = 4-8/group). **(H)** Representative μCT images of knees injected with Lpl1 (10 μg/knee) from NMRI mice depleted of neutrophils or monocytes/macrophages on day 7. **(I)** Synovitis severity induced by i.a. knee injection of 20 μl of Lpl1 (0.33 μg/knee) on day 3 in NMRI mice treated with either etanercept, a tumor necrosis factor inhibitor (anti-TNF) or anakinra, an interleukin 1 receptor antagonist (anti-IL1) (n = 9-12/group). **(J)** Differences in knee joint swelling (mm) and **(K)** frequency of bone erosion based on a μCT scan between C57BL/6 wild-type (WT) and TLR2-deficient (TLR2-/-) mice on day 7 after i.a. injection of Lpl1 (20 μg/knee, n = 4-5/group). Statistical evaluations were performed using the Mann–Whitney U test, with data expressed as the mean ± standard error of the mean (A, B, C, E, F, G, I, and J), or Fisher’s exact test (K). *P < 0.05; **P < 0.01; ***P < 0.001.

Next, to elucidate which immune cells were responsible for the onset of arthritis, mice depleted of monocytes/macrophages, neutrophils or T-cells were i.a. injected with 0.33 μg Lpl1, and the severity of the histopathological synovitis was examined on day 3. The depletion of synovial macrophages and infiltrating monocytes by clodronate liposomes significantly reduced the severity of the synovitis (Fig 3A), whereas neutrophil depletion by Ly6G antibodies had no effect on synovitis severity (Fig 3B). To investigate the role of T-cells, mice were simultaneously depleted of CD4 and CD8 T-cells by intraperitoneal injection of anti-CD4 and anti-CD8 antibodies. No notable difference regarding the severity of synovitis was observed between the groups (Fig 3C). Additionally, CTLA-Ig treatment (abatacept) that blocks T-cell activation had no effect on synovitis development, suggesting that T-cells were not essential for acute Lpp-induced joint inflammation. Fig 3D represents a typical picture of the knee joint from mice depleted of monocytes/macrophages showing the absence of leukocyte infiltration.

To examine which cells were responsible for causing bone erosion, a higher dose of Lpl1 (10 μg/knee) was i.a. injected. In line with the data described above, depletion of macrophages significantly attenuated macroscopic inflammation (Fig 3E) and more strikingly, completely protected the joints from bone damage caused by Lpl1 (Fig 3F and 3H). No difference was found regarding bone erosions between the controls and neutrophil-depleted mice (Fig 3G and 3H).

TNF-α and IL-1, released by monocytes/macrophages, are known to play a crucial role in septic arthritis [10, 12, 22]. To study the role of these cytokines in Lpl1-induced synovitis, both anti-TNF treatment (etanercept) and anti-IL1 treatment (anakinra) [10, 11] were used. Anti-TNF, but not anti-IL1, treatment significantly reduced the synovitis severity (Fig 3I) compared to the severity in the PBS-treated controls, indicating that Lpl1-induced arthritis is partially mediated by TNF-α.

Finally, to study whether joint destruction caused by Lpp, a major ligand for TLR2 [23, 24], is mediated through this receptor, TLR2^-/-^ mice were i.a. injected with Lpl1. The TLR2^-/-^ mice barely developed clinical signs of arthritis (Fig 3J), and no joint destruction was observed compared to that of the wild-type mice, of which 100% presented joint erosions (Fig 3K).

### Deficiency of prelipoprotein lipidation in *S*. *aureus* results in bacterial immune evasion

**Fig 4:**
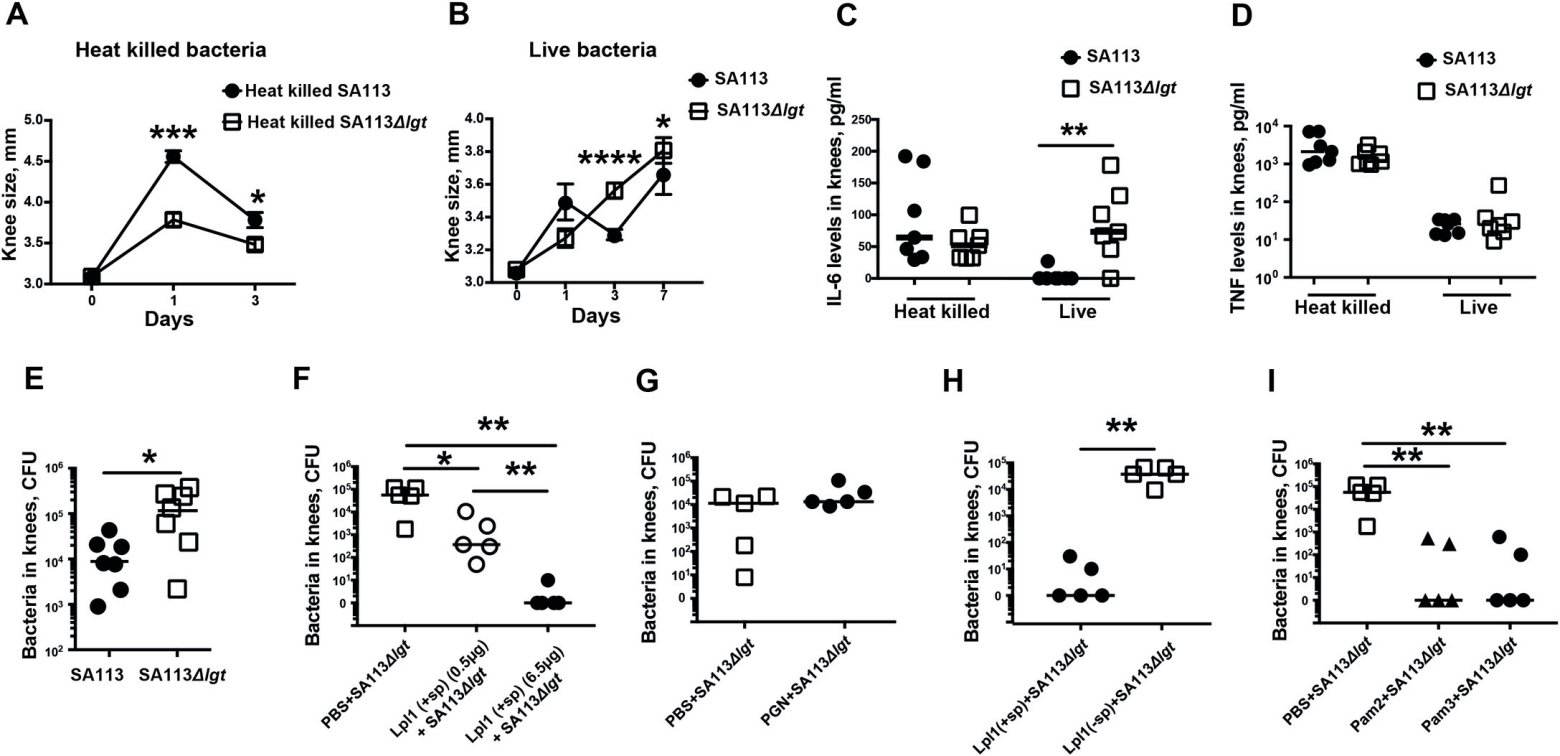
Lipoprotein deficiency in *S*. *aureus* results in bacterial overgrowth, and coinjection of purified lipoprotein with *S*. *aureus* leads to bacterial elimination in mouse knee joints. Measurement of knee swelling (mm) in NMRI mice following intra-articular (i.a.) knee injection of 20 μl of **(A)** heat-killed *S*. *aureus* SA113 or SA113*Δlgt* mutant (2 x 10^8^ CFU/knee) (n = 7-11/group), or **(B)** live *S*. *aureus* SA113 or SA113*Δlgt* mutant (1 x 10^4^ CFU/knee) (n = 7-21/group). **(C)** IL-6 and (**D**) TNFα levels in tissue homogenates of knees injected with either heat-killed or live *S*. *aureus* SA113 or SA113*Δlgt* mutant on day 3 (n = 7/group). **(E)** Bacterial counts of *S*. *aureus* in the knee joints of the mice 3 days after i.a. infection of SA113 or SA113*Δlgt* mutant (1 x 10^4^ CFU/knee, n = 7/group). Bacterial counts in mouse knee joints 3 days after i.a. coinjections of *S*. *aureus* SA113*Δlgt* (1–1.4 x 10^4^ CFU/knee) **(F)** with different doses of purified *S*. *aureus* lipoprotein, denoted as Lpl1(+sp) (0.5 or 6.5 μg/knee) or PBS (n = 5/group); (**G**) with *S*. *aureus* peptidoglycan (PGN) (5 μg/knee) or PBS (n = 5/group); **(H)** with lipidated (Lpl1(+sp)) or unlipidated Lpl1 protein (Lpl1(-sp)) (5 μg/knee, n = 5/group); or **(I)** with lipopeptides (Pam2CSK4 and Pam3CSK4; 2 μg/knee, n = 5/group). Statistical evaluations were performed using the Mann–Whitney U test, with data expressed as the mean ± standard error of the mean (A, B), or median (C-I). *P < 0.05; **P < 0.01, ***P < 0.001; ****P < 0.0001.

To understand the role of Lpps in *S*. *aureus* septic arthritis, both heat-killed and live *S*. *aureus* SA113 strains, as well as a deletion mutant of the SA113 strain lacking *lgt*, were inoculated into the mouse knee joints. The heat-killed SA113*Δlgt* mutant strain resulted in less joint swelling than the SA113 parental strain on days 1 and 3 (Fig 4A). Surprisingly, the reverse phenomenon was observed when live bacteria were used. The SA113*Δlgt* mutant strain caused a significantly higher degree of joint swelling on days 3 and 7 than the arthritis caused by its parental strain (Fig 4B). The discrepancy between the heat-killed and live *S*. *aureus* SA113*Δlgt* mutant strains was also observed with regard to IL-6 levels in the knee homogenates on day 3. Higher levels of IL-6 were induced by the live SA113*Δlgt* mutant, and a tendency towards lower levels of IL-6 was induced by the heat-killed SA113*Δlgt* mutant compared to the parental SA113 strain (Fig 4C), whereas no differences were seen with regard to TNF-α levels (Fig 4D). This unexpected increase in joint swelling and IL-6 levels could be explained by the higher bacterial load found in the knee joints of the SA113*Δlgt* mutant-inoculated mice on day 3 (Fig 4E).

To further elucidate the mechanism whether SA113*Δlgt* mutant survived better in joints than its parental stain, we analyzed the cytotoxicity of those strains towards mouse splenocytes (S2 Fig). No significant difference was observed between those two strains, demonstrating that SA113*Δlgt* mutant does not have enhanced killing capacity of immune cells compared to its parental strain. We speculate that SA113*Δlgt* mutant is more resistant to immune-mediated bacterial killing than its parental stain. We reasoned that this might be due to a lack of Lpps that trigger the immune response.

### Coinjection of lipoproteins and live *S*. *aureus* drastically increases bacterial elimination in a dose- and lipid-moiety-dependent manner

To test the above hypothesis, live *S*. *aureus* SA113*Δlgt* mutants mixed with various concentrations of Lpl1(+sp), Lpl1(-sp), or PBS were i.a. injected into mouse knee joints. Strikingly, the bacterial load in the knee joints was dose-dependently reduced when SA113*Δlgt* mutant was simultaneously administered with Lpl1(+sp). For the Lpp dose of 6.5 μg/knee, only 1 out of 5 knee joints was positive for bacterial culture with very low bacterial counts (10^1^ colony forming units (CFUs)/knee), whereas in the PBS controls, all knee joints had high CFU counts (10^5^ CFU/knee) on day 3 postinfection (Fig 4F). In contrast, a comparable dose (5 μg/knee) of PGN purified from the SA113*Δlgt* mutant completely lacked the capacity to induce an eradicating effect on the bacteria (Fig 4G). Importantly, the bacterial elimination effect was completely abolished when Lpl1(-sp), lacking the lipid moiety, was used (Fig 4H). Additionally, synthetic lipopeptides (Pam2CSK4 and Pam3CSK4) exhibited similar bacterial elimination effects as the Lpps, indicating that the lipid moiety of Lpl1 is important for bacterial elimination (Fig 4I).

### The association between arthritis severity, bacterial load, and release of neutrophil attracting chemokines at the early course of *S*. *aureus* septic arthritis

**Fig 5:**
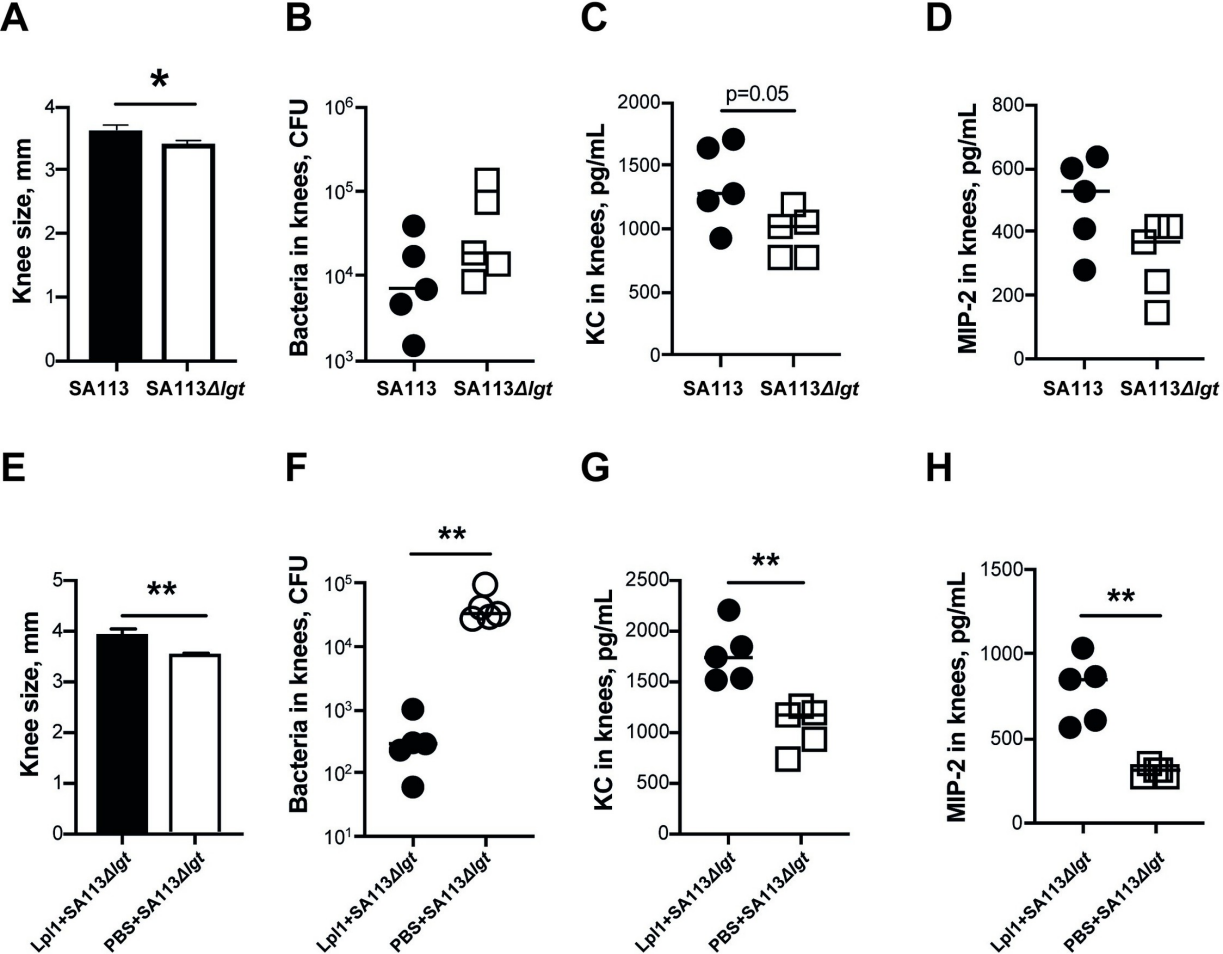
The association between arthritis severity, bacterial load, and release of neutrophil attracting chemokines at the early course of *S*. *aureus* septic arthritis. Measurement of knee swelling (mm) (**A**), bacterial counts in mouse knees (**B**), the levels of keratinocyte chemoattractant (KC) (**C**), and macrophage inflammatory protein-2 (MIP-2) (**D**) in knees from NMRI mice receiving intra-articular (i.a.) injection of 20 μl of *S*. *aureus* SA113 or SA113*Δlgt* mutant (1 x 10^4^ CFU/knee) on day 1 postinfection (n = 5/group). Measurement of knee swelling (mm) (**E**), bacterial counts in mouse knees (**F**), the levels of KC (**G**) and MIP-2 (**H**) in knees from NMRI mice receiving i.a. coinjection of 20 μl of *S*. *aureus* SA113*Δlgt* mutant (1.4 x 10^4^ CFU/knee) with either purified staphylococcal lipoprotein, denoted as Lpl1(+sp) (5 μg/knee) or PBS on day 1 postinfection (n = 5/group). Statistical evaluations were performed using the Mann–Whitney U test, with data expressed as the mean ± standard error of the mean (A, E), or median (B-D, F-H). *P < 0.05; **P < 0.01.

To provide the evidence to support our hypothesis that SA113*Δlgt* mutant arouses immune responses to a lesser extent than its parental strain, we i.a inoculated both bacterial strains into mouse knee joints and compared the knee sizes, CFU counts in joints, and the levels of neutrophil attracting chemokines (KC and MIP-2) on day 1 postinfection. The joints inoculated with SA113 strain were significantly more swollen (Fig 5A) and tended to have lower bacterial counts (Fig 5B) compared to SA113*Δlgt* injected knees. However, higher levels of KC were detected in SA113 injected knees than SA113*Δlgt* injected knees (Fig 5C). Similar trend was also found in MIP-2 levels (Fig 5D). These results demonstrate that SA113*Δlgt* is less potent to induce neutrophil attracting chemokines in *S*. *aureus* septic arthritis compared with its parental strain.

We further analyzed the effect of addition of exogenous Lpl1 to SA113*Δlgt* in the similar setting. Strikingly, addition of Lpl1 to SA113*Δlgt* resulted in increased joint swelling (Fig 5E), decreased CFU counts (Fig 5F), and higher levels of KC and MIP-2 (Fig 5G and 5H) in the knees on day 1 postinfection.

### The enhanced bacterial killing effect triggered by lipoproteins is mediated by neutrophils and monocytes/macrophages through TLR2

**Fig 6:**
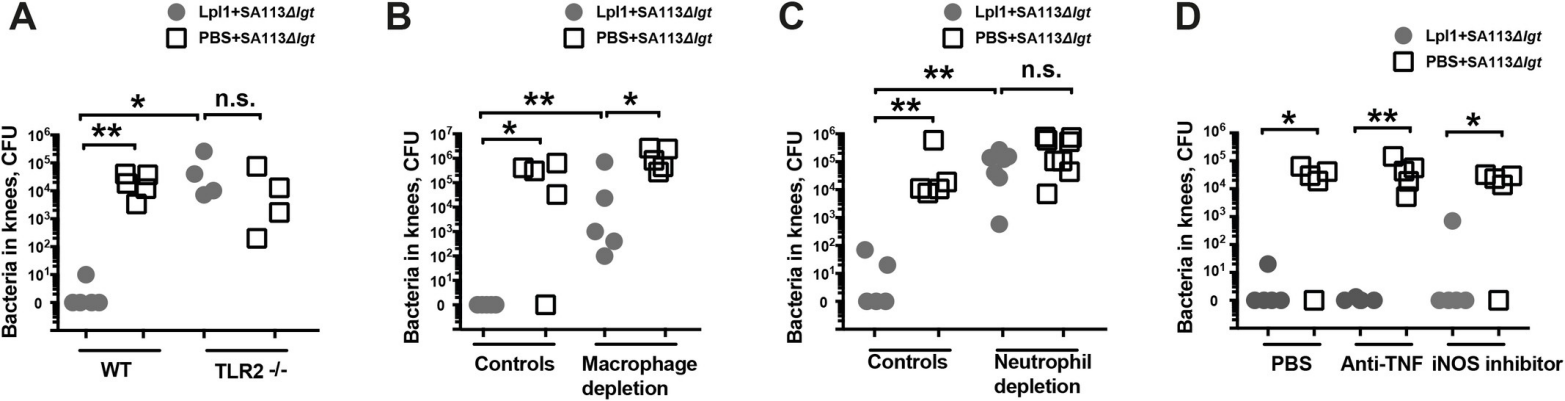
TLR2, monocytes/macrophages, and neutrophils significantly contribute to the bacterial killing effect induced by lipoproteins. Bacterial counts in mouse knee joints 3 days after intra-articular (i.a.) coinjections of *S*. *aureus* SA113*Δlgt* mutant (1.4 x 10^4^ CFU/knee) with either PBS or purified staphylococcal lipoprotein, denoted as Lpl1(+sp) (5 μg/knee) **(A)** in C57BL/6 wild-type (WT) and TLR2-deficient (TLR2-/-) mice (n = 4-5/group); **(B)** in NMRI mice depleted of monocytes/macrophages using clodronate liposomes (n = 5/group); **(C)** in NMRI mice depleted of neutrophils using anti-Ly6G antibody (n = 5-8/group); and **(D)** in NMRI mice treated with either etanercept, a tumor necrosis factor inhibitor (anti-TNF) or 1400W, an iNOS inhibitor or PBS (n = 5/group). Statistical evaluations were performed using the Mann–Whitney U test. *P < 0.05; **P < 0.01, n.s. = not significant.

To understand the mechanism by which *S*. *aureus* Lpps mediate bacterial killing, we first studied whether the Lpps possess bactericidal capacity. Our data suggest that neither Lpp nor lipopeptides had direct bactericidal effect since *in vitro* incubation of SA113*Δlgt* with Lpl1 or Pam3CSK4 did not affect bacterial proliferation or survival (S3 Fig). Since Lpps are specific ligands for TLR2 [23, 24], we hypothesized that the protective effect of Lpps is mediated by TLR2. Indeed, the bacterial elimination induced by Lpl1 was completely abolished in TLR2^-/-^ mice (Fig 6A), strongly suggesting that TLR2 is essential for Lpp-induced bacterial killing.

Next, we studied the importance of monocytes/macrophages and neutrophils in the knee joints after Lpl1 injection. The knee joints of monocyte/macrophage-depleted mice injected with a mixture of Lpl1 and SA113*Δlgt* mutant exhibited significantly higher bacterial counts than the knee joints of the non-depleted mice (Fig 6B). However, macrophage depletion failed to fully abolish the effect of Lpps on bacterial elimination, suggesting that other cell types also contributed to the effect. Importantly, the protective effect of the Lpps completely disappeared in the neutrophil-depleted mice (Fig 6C), suggesting that Lpl1 elicits neutrophils to kill bacteria. We further studied whether Lpps boost the phagocytic capacities of phagocytes *in vitro*. Mouse peritoneal macrophages stimulated with Lpl1 were incubated with green fluorescent protein (GFP)-expressing *S*. *aureus*. The *S*. *aureus* internalization rates in macrophages were analyzed by flow cytometry imaging. As expected, the opsonization of bacteria with mouse sera resulted in a 3–4 times higher rate of phagocytosis. However, no notable differences were observed between the Lpl1-stimulated and non-stimulated groups (S4 Fig). Furthermore, the whole blood killing assay was thereafter performed to verify whether SA113*Δlgt* mutant survived better in whole blood than its parental strain. No tangible difference was observed between groups (S5 Fig).

As Lpps induced TNF-α release by macrophages (S6 Fig) and anti-TNF treatment attenuated the severity of Lpp-induced arthritis (Fig 3I), we posed the question of whether the bacterial eliminating effect of Lpp was TNF-dependent. Coinjection of Lpl1 and *S*. *aureus* SA113*Δlgt* mutant reduced bacterial loads in a similar manner in mice treated with anti-TNF drug as in mice receiving PBS treatment (Fig 6D), suggesting that TNF is not involved in Lpl1-mediated bacterial killing. *S*. *aureus* Lpps are known to induce nitric oxide production by macrophages [25], and the expression of inducible nitric oxide synthase (iNOS) is associated with protective immunity against bacterial infections [26]. We used an iNOS inhibitor (1400W) to block iNOS activity in mice receiving a mixture of Lpl1 and the SA113*Δlgt* strain. No notable difference was observed between the PBS-treated and iNOS inhibitor-treated animals (Fig 6D).

### Quick release of neutrophil chemoattractants by macrophages is responsible for the lipoprotein-induced protective effect

**Fig 7:**
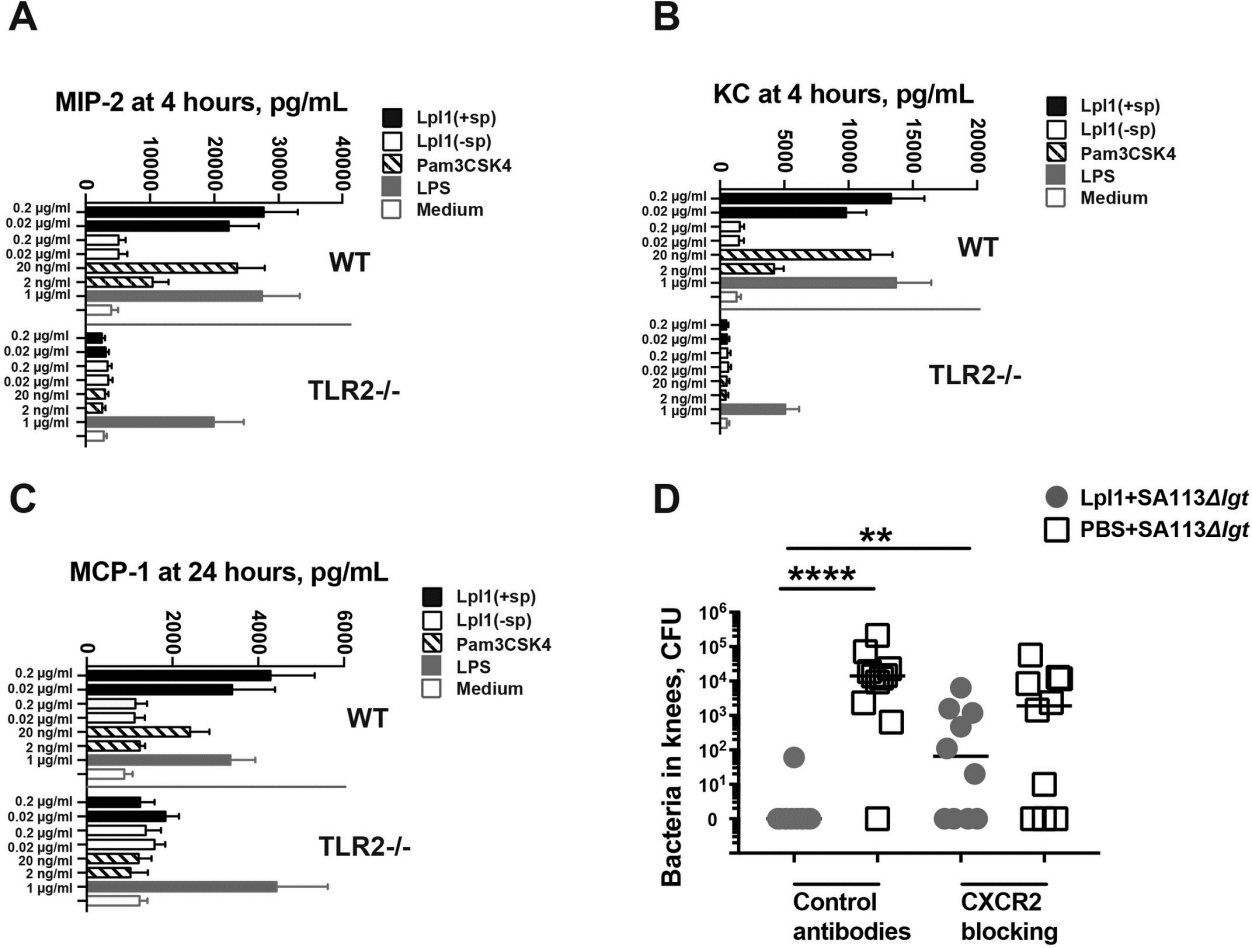
Neutrophil-recruiting chemokines released by macrophages upon lipoprotein stimulation mediate the bacterial killing effect. The levels of **(A)** macrophage inflammatory protein-2 (MIP-2), **(B)** keratinocyte chemoattractant (KC), and **(C)** monocyte chemoattractant protein 1 (MCP-1) in the supernatants collected from C57BL/6 wildtype (WT) and TLR-2 deficient (TLR2-/-) (n = 4/group) mouse peritoneal macrophage cell cultures (5x10^5^ cells/mL) after stimulation with purified staphylococcal lipoprotein, denoted as Lpl1(+sp) (0.02–0.2 μg/ml); unlipidated Lpl1 protein, denoted as Lpl1(-sp) (0.02–0.2 μg/ml); Pam3CSK4 (2–20 ng/ml); lipopolysaccharide (LPS) (1 μg/ml); or culture medium for 4 or 24 hours. **(D)** Bacterial counts in mouse knee joints 3 days after intra-articular coinjections of *S*. *aureus* SA113*Δlgt* mutant (1.4 x 10^4^ CFU/knee) with either PBS or purified staphylococcal lipoprotein, denoted as Lpl1(+sp) (5 μg/knee) in NMRI mice treated with CXCR2 blocking antibodies and isotype control antibodies (n = 10-11/group). Statistical evaluations were performed using the Mann–Whitney U test, with data expressed as the mean ± standard error of the mean (A-C) or median (D). ** *P* < 0.01, **** *P* < 0.0001.

Since macrophages, but not neutrophils, are present in the synovium of healthy joints and monocytes/macrophages were responsible for the Lpp-induced joint inflammation (Fig 2 and 3), we hypothesized that Lpl1 stimulates macrophages via TLR2, resulting in the release of large amounts of chemokines that in turn recruit neutrophils to kill bacteria. Indeed, peritoneal macrophages from wild-type mice stimulated with Lpl1 exhibited a quick dose-dependent release of neutrophil chemoattractant MIP-2 and KC 4 hours after stimulation and the monocyte-attracting chemokine MCP-1 24 hours after stimulation, whereas macrophages from TLR2^-/-^ mice displayed no such chemokine release (Fig 7A–7C). Mouse splenocytes, mainly composed of B- and T-lymphocytes, produced neither MIP-2 nor KC upon Lpl1 stimulation at 4 hours nor MCP-1 at 24 hours in wild-type and TLR2^-/-^ mice. After 24 hours of stimulation, TNF-α levels were elevated in the supernatants of both peritoneal macrophages and splenocytes from wild-type mice stimulated with Lpl1(+sp) and Pam3CSK4 but not those stimulated with Lpl1(-sp) (S6 Fig). As expected, increased TNF-α was observed in only LPS-stimulated cells from TLR2^-/-^ mice.

To further elucidate the importance of neutrophils attracting chemokine release in the Lpp-induced protective effect, CXCR2 blocking antibodies were used to inhibit *in vivo* neutrophil chemotaxis. Indeed, CXCR2 blocking antibodies efficiently reduced the total number of infiltrating neutrophils in Lpl1-injected knee joints to 13% on day 3. Importantly, decreasing the infiltrating neutrophils by CXCR2 blocking antibodies almost fully abrogated the effect of bacterial elimination induced by Lpl1 (Fig 7D), strongly suggesting that the release of neutrophil-attracting chemokines upon Lpp stimulation is the key mechanism of Lpp-induced bacterial killing.

### Lipoproteins attenuate *S*. *aureus*-induced septic arthritis

**Fig 8:**
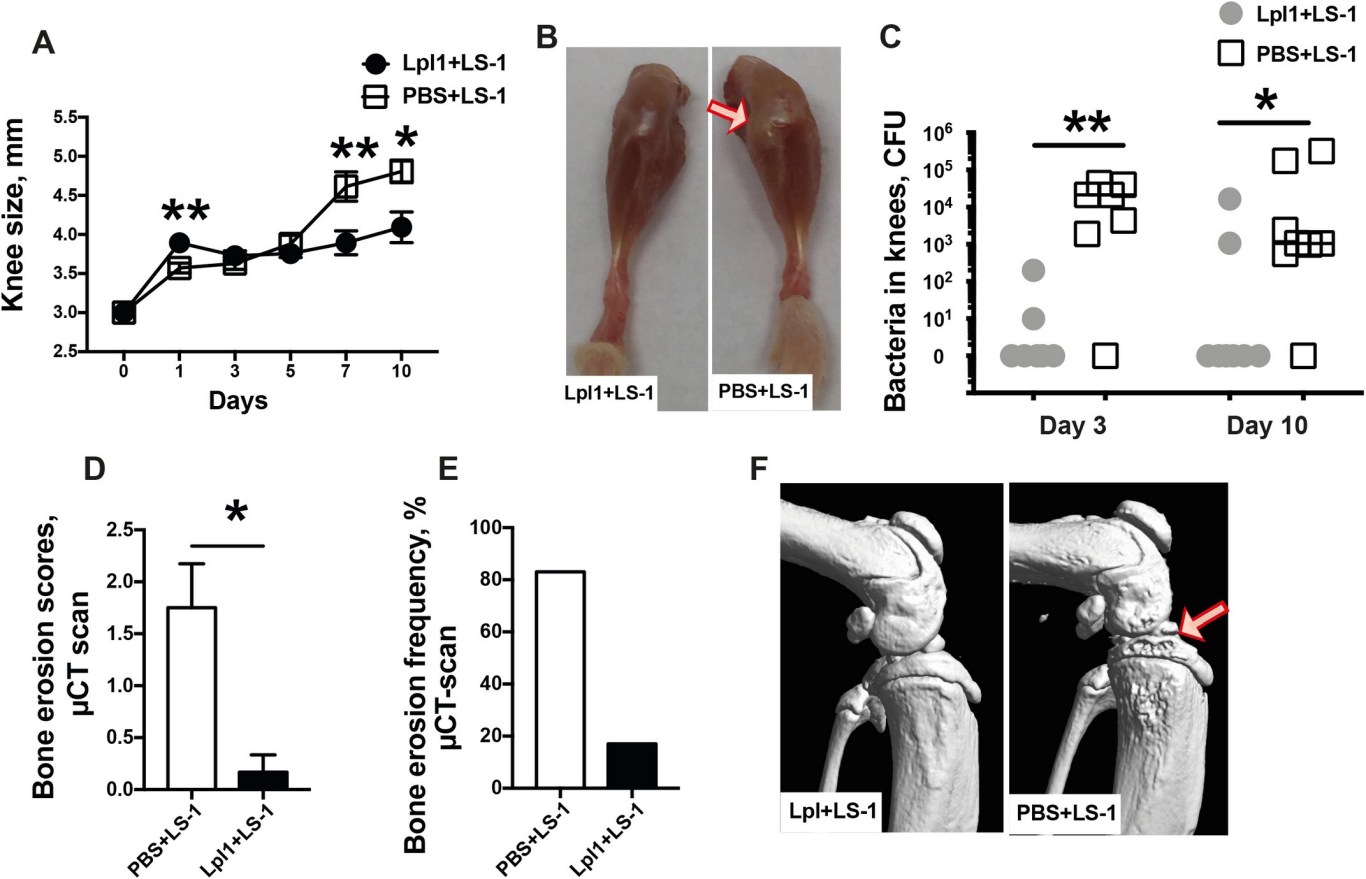
Coinjection of staphylococcal lipoproteins with *S*. *aureus* LS-1 attenuates septic arthritis. **(A)** Measurement in knee swelling (mm) and **(B)** representative images of knees from NMRI mice on day 10 after intra-articular (i.a.) coinjections of 20 μl of *S*. *aureus* LS-1 strain (4 x 10^3^ CFU/knee) with either purified *S*. *aureus* lipoprotein Lpl1 (5 μg/knee) or PBS (n = 14/ group). The arrow indicates synovitis. **(C)** Bacterial counts in mouse knee joints 3 days and 10 days after infection with the *S*. *aureus* LS-1 strain (4 x 10^3^ CFU/knee) with either Lpl1 (5 μg/knee) or PBS (n = 7-8/group). The bone erosion scores **(D)** and bone erosion frequency **(E)** in mouse knee joints (n = 6/group) as evaluated by a μCT scan and **(F)** representative μCT images from knee joints injected with the *S*. *aureus* LS-1 strain and Lpl1 mixture (left panel) or with the *S*. *aureus* LS-1 strain and PBS mixture (right panel) on day 10. The arrows indicate bone erosion. Statistical evaluations were performed using the Mann–Whitney U test, with data expressed as the mean ± standard error of the mean (A, D) or median (C), or Fisher’s exact test (E). *P < 0.05 and **P < 0.01.

We next investigated whether enhanced bacterial elimination induced by Lpps leads to a better clinical outcome in septic arthritis. Mice were inoculated i.a. with a mixture of Lpl1 and the *S*. *aureus* LS-1 strain that was originally isolated from a mouse that spontaneously developed septic arthritis [21]. Mice injected with a mixture of Lpl1 and LS-1 had less swelling in their knee joints on days 7 and 10 postinfection (Fig 8A and 8B) and less bacterial load in their joints than mice in the control group that received a mixture of PBS and LS-1 (Fig 8C). Furthermore, these mice exhibited significantly less pronounced bone erosions with lower frequency than the mice in the control group (Fig 8D–8F), suggesting that the bacterial eradication effect elicited by Lpl1 applies not only to the *S*. *aureus* mutant strain deficient in lipidation of prelipoproteins but also to the wildtype *S*. *aureus* strains.

## Discussion

Our results demonstrate the dual role of staphylococcal Lpp in *S*. *aureus*-induced septic arthritis. On the one hand, Lpps induce inflammatory reactions and joint destruction mediated by monocytes/macrophages. On the other hand, Lpps cause the quick release of chemokines and consequent neutrophil recruitment, resulting in efficient bacterial killing. Importantly, both the lipid moiety of Lpps and TLR2 were identified as the molecular structures responsible for this outcome.

Postinfectious complications in septic arthritis, such as joint deformation and deleterious contractures, remain a major medical challenge. Exaggerated immune responses have been proposed as the cause of such complications [9, 12]. There are many bacterial components of *S*. *aureus* that possess the capacity to induce joint inflammation [12, 27, 28]. However, it is still unclear which component is most important in real-life infections. To address this uncertainty, not only the capacity of immune stimulation but also the quantity of components expressed in a single bacterium should be taken into consideration. A single intra-articular injection of Lpp (10 μg/knee) induced chronic macroscopic arthritis lasting for at least 3 weeks as well as severe joint destruction verified by both histopathological and radiological examinations, demonstrating that Lpp is an arthritogenic molecule. Lpps were highly potent since they exerted a strong immunostimulatory effect even at the nanogram level *in vivo*. More importantly, the mice injected with the SA113*Δlgt* mutant strain lacking lipidation displayed less severe joint swelling than the mice injected with the SA113 parental strain at the early time points before the bacterial proliferation of *Δlgt* mutants exceeded that of the parental strains. This finding strongly suggests that Lpps are one of the major arthritogenic bacterial components. However, we have to keep in mind that the *Δlgt* strain that lacks lipidation maintained the capacity to induce joint inflammation, indicating that Lpps are not the only molecule in *S*. *aureus* that cause joint inflammation. Although intra-articular injection of PGN (10 μg/knee) induced only transient and milder joint swelling, the importance of PGN in arthritis induction cannot be ruled out since PGN is the most abundant molecule in *S*. *aureus*. The synergistic effect of PGN and lipopeptides in immune activation has been reported previously [29].

Our data compellingly demonstrate that both neutrophils and monocytes rapidly migrated into the joints injected with Lpps, and monocytes/macrophages were fully responsible for joint destruction in Lpp-induced arthritis. Focal bone destruction in autoimmune arthritis is due to excessive bone resorption resulting from osteoclast activation that is mediated by local expression of receptor activator of nuclear factor kappa-B (RANKL) that is higher than that of its decoy receptor osteoprotegerin (OPG)[30]. Osteoclasts not only exist inside of the bone but also can be derived from mature monocytes and macrophages when a suitable microenvironment is provided by bone marrow-derived stromal cells [31], which might be the case in this study. In fact, monocytes/macrophages were shown to mediate bone erosion in arthritis induced by other *S*. *aureus* components, such as bacterial DNA [27] and peptidoglycan [28], as well as antibiotic-killed *S*. *aureus* [12], suggesting that monocytes/macrophages might be the most important immune cells that determine the progression of septic arthritis. Indeed, mice depleted of monocytes developed less severe septic arthritis and joint lesions despite decreased bacterial clearance and higher mortality [32].

Macrophages are a major source of many cytokines involved in the immune response. In autoimmune arthritic diseases, proinflammatory cytokines play an important pathogenetic role. In particular, TNF release by macrophages, fibroblasts and T-cells in inflamed synovial tissue leads to joint swelling and subsequent joint destruction [33]. Furthermore, a previous study showed that the blockade of TNF, but not IL-1, resulted in a reduction of bone erosions in a murine TNF-driven arthritis model [34]. Previously, we have shown that antibiotic-killed *S*. *aureus* induces arthritis through the TNF receptor. In the present study, TNF also played an important role in Lpp-induced arthritis since a) Lpps induced TNF release in both macrophages and splenocytes and b) TNF inhibition, but not IL-1 inhibition, significantly reduced synovitis severity.

Unexpectedly, the live *Δlgt* strain gave rise to significantly more severe joint inflammation, although the heat-killed *Δlgt* strain caused less synovitis. This result is due to the fact that the *Δlgt* strain is more tolerant to host immune-mediated bacterial killing than the parental strain, as the bacterial load of the SA113 parental strain in the knee joints was significantly lower than that of the *Δlgt* strain. This finding is in line with the previous report that revealed that a lack of *S*. *aureus* Lpps causes bacterial immune evasion and lethal infections with disseminated abscess formation in mice with systemic *S*. *aureus* infection [35]. The importance of the lipid moiety in bacterial elimination was further confirmed by our experiments when Lpps were coinjected with *S*. *aureus* into mouse knee joints. This situation is a perfect example of the dual sides of host responsiveness to bacterial infections—on one hand, the host response protects the host against bacteria, but on the other hand, the response sometimes increases the infection severity when danger signals trigger exaggerated host responses.

How is the bacterial killing effect mediated by Lpps? Lpps themselves had no direct effect on bacterial proliferation. Rather, the enhanced local immune response triggered by Lpps was responsible for the bacterial killing effect. Macrophages, neutrophils and natural killer (NK) cells are the most important immune cell types in innate immunity. NK cells can be activated and exert their biological functions upon stimulation by synthetic lipopeptides with sequences from Lpps of *S*. *aureus* [36]. Macrophages seem to play a partial role, as the indirect bacterial killing effect was diminished in mice depleted of macrophages/monocytes. This finding suggests that Lpps have the capacity to trigger the activation of macrophages to better control and eliminate bacteria. Macrophages are phagocytes that play an important role in *S*. *aureus* septic arthritis [32]. *S*. *aureus* Lpps are the major inducers of inducible nitric oxide synthase (iNOS) and nitric oxide (NO) production in mouse macrophages [25]. The production of nitric oxide is important in controlling bacterial infections [26, 37]. However, treatment with an iNOS inhibitor had no effect on the bacterial eradication induced by Lpps, suggesting that NO production is not critical for Lpp-induced bacterial killing. Notably, no complete abrogating effect was observed as a result of macrophage depletion, which indicates the involvement of other immune cells. Neutrophils were the most predominant cell type that infiltrated the local joints upon Lpp stimulation. Additionally, the total abrogation of the bacterial killing effect by Lpps in neutrophil-depleted mice strongly suggests that neutrophils are fully responsible for the protective effect of Lpps. In healthy joints, there are a very limited number of neutrophils. For the neutrophils to reach the joint cavity and exert their biological function, neutrophil chemoattractants are needed. KC and MIP-2 are known major chemokines responsible for recruiting neutrophils, and both bind to CXCR2 [38]. Resident tissue macrophages have been shown to be the major source of neutrophil chemokines [39]. In the current study, macrophages, but not T- or B-cells, quickly released large amounts of neutrophil-recruiting chemokines upon Lpp stimulation, and such chemokine release was fully controlled by lipid moiety-TLR2 signaling. Indeed, a positive feedback loop may exist in Lpp-injected knee joints, as monocytes are recruited to the local joints where they probably respond to Lpp stimulation with chemokine release that leads to an additional influx of monocytes and neutrophils to the local inflammation site. Actually, the bacterial killing effect of phagocytes might also be increased by Lpps, as Lpps can directly activate neutrophils by upregulating CD11b/CD18 and enhancing the production of reactive oxygen species [40].

In recent years, immune therapy targeting negative regulators of immune activation (immune checkpoints) has been an exciting research area in drug development, with promising results achieved in patients with a variety of cancers [41]. Similarly, in infectious diseases, the activation of the immune system to eliminate invading bacteria has always been an ultimate goal to overcome the challenges caused by the rapid emergence of antibiotic-resistant bacteria. The development of a vaccine that prevents *S*. *aureus* infections is of interest. However, thus far, all attempts to develop active or passive immunization against *S*. *aureus* have failed, which might be due to the lack of a well-defined, single virulence factor in *S*. *aureus* [42]. Purified Lpp acted in a protective fashion by means of less radiological bone destruction after treatment with a mixture containing a known pathogenic staphylococcal strain, i.e., LS-1. Our results suggest that Lpps/lipid moieties might be used as a potential candidate for immune therapy to combat local *S*. *aureus* infections, e.g., septic arthritis and osteomyelitis. In this case, the dose of Lpps should be carefully controlled to keep the local tissue damage and efficient bacterial killing effect in balance. In addition, a similar protective immune response might also be induced by staphylococcal Lpps to eradicate other invading pathogens, such as gram-negative bacteria or even antibiotic-resistant bacteria.

In conclusion, intra-articular injection of staphylococcal Lpps induced chronic joint inflammation and bone erosion. The observed effect was mediated by macrophages via TLR2 signaling and partially involved TNF. Surprisingly, purified *S*. *aureus* Lpps strengthened the immune response, consequently reducing bacterial burden and attenuating bone destruction in septic arthritis. Our findings may pave the way for the development of a novel strategy to address the challenge of antibiotic resistance.

## Materials and methods

### Ethics statement

Mouse studies were reviewed and approved by the Ethics Committee of Animal Research of Gothenburg (Ethical number 58–2015). Mouse experiments were conducted in accordance with recommendations listed in the Swedish Board of Agriculture's regulations and recommendations on animal experiments.

### Mice

Female NMRI mice and C57Bl/6 wild-type mice of both sexes, aged 6–8 weeks, were purchased from Envigo (Venray, Netherlands) and Charles River Laboratories (Sulzfeld, Germany), respectively. Toll-like receptor 2-deficient B6.129-Tlr2^tm1Kir^/J (TLR2^-/-^) mice of both sexes were purchased from The Jackson laboratory (Bar Harbor, Maine, USA). All mice were housed in the animal facility of the Department of Rheumatology and Inflammation Research, University of Gothenburg. Mice were kept under standard temperature and light conditions and were fed laboratory chow and water ad libitum.

### Preparation of bacterial solutions

The SA113, SA113*Δlgt* mutant[15], and LS-1 [43] *S*. *aureus* strains were prepared as described. The strains were stored at -70°C until use. Before the experiments, the bacterial solutions were thawed, washed with sterile PBS, and adjusted to the required concentration.

For experiments with heat-killed bacteria, the SA113 and SA113*Δlgt* mutant *S*. *aureus* strains were heat-killed at 95°C for 45 min and thereafter adjusted to a similar concentration using optical density at 600 nm (OD_600_). To ensure that no bacteria survived, the bacterial suspensions were plated and cultured for 24 hours. No bacterial growth was detected.

### Expression and purification of Lpl1(+sp) and Lpl1(–sp)

The preparation and purification of the *S*. *aureus* lipoproteins Lpl1(+sp) and Lpl1(-sp) were performed by Dr. Nguyen (Microbial Genetics, University of Tübingen, Germany), as previously described [20]. Lpl1(+sp) was isolated from the membrane fraction of *S*. *aureus* SA113 (pTX30::*lpl*1-his), whereas Lpl(-sp) was isolated from the cytoplasmic fraction of *S*. *aureus* SA113*Δlgt* (pTX30::*lpl*1(-sp)-his). Both of these Lpl1-his proteins were purified via Ni-NTA affinity chromatography. For the enhancement of protein expression, the clones were first cultivated aerobically at 37°C in the absence of xylose (BO-medium) until OD578 nm ≈ 0.5 was reached and were thereafter continuously cultivated for 4 hours in the presence of 0.5% xylose to induce Lpl1 expression. The bacterial cells were harvested and washed twice with Tris buffer (20 mM Tris, 100 mM HCl, pH 8.0). Then, the pellet was resuspended in Tris buffer containing a protease inhibitor tablet (Merck, Darmstadt, Germany) and lysostaphin (30 μg/ml) and was incubated at 37°C for 2 hours to disrupt the cell wall. After the first ultracentrifugation (235,000 x g for 45 min at 4°C), the supernatant containing the cytoplasmic proteins was collected for the next purification step. For membrane fraction isolation, the pellet was subsequently dissolved overnight at 6°C with Tris buffer containing 2% Triton-X100. After the second ultracentrifugation step, the supernatant containing membrane fragments was collected. The purification step was carried with Ni-NTA Superflow beads (Qiagen, Germany). The Ni-NTA beads capturing Lpl1 proteins were washed four times with the first washing buffer (Tris buffer containing 0.25% Triton X-100 and 20 mM imidazole), followed by washing 2 times with the second washing buffer (Tris buffer containing 0.25% Triton X-100 and 40 mM imidazole). Finally, Lpl1 was eluted with Tris buffer containing 500 mM imidazole. The Lpl1 were concentrated via a centrifugal ultrafilter unit with a molecular mass cut-off of 10 kDa (Sartorius AG, Göttingen, Germany). The concentrated samples of Lpl1 were dialyzed overnight at 6°C with Dulbecco’s PBS (DPBS) buffer (Life Technologies, Darmstadt) by a MWCO 6–8 kDa tube dialyzer (Merck, Darmstadt) and were subsequently lyophilized overnight. A total of 2 μg of lyophilized samples were dissolved in water and subjected to sodium dodecyl sulfate-polyacrylamide gel electrophoresis (SDS-PAGE) to determine the purity and quantity of purified protein samples. The purified compounds of Lpl1 were stored at -70°C until use and were adjusted in PBS to the required concentration before each experiment.

### Experimental protocols for the induction of arthritis with staphylococcal lipoproteins

To study the arthritogenic properties of *S*. *aureus* Lpps, six sets of experiments were performed, and NMRI, C57Bl/6 wild-type or TLR2^-/-^ mice were intra-articularly injected into the knee joint with one of the following compounds in 20 μl of PBS: 1) purified Lpl1(+sp) or Lpl1(-sp) *S*. *aureus* Lpps; 2) heat-killed SA113 or SA113*Δlgt* mutant *S*. *aureus* strains; 3) live SA113 or SA113*Δlgt* mutant *S*. *aureus* strains; 4) solutions containing mixtures of SA113*Δlgt* mutant with either Lpl1(+sp), Lpl1(-sp), PGN or two synthetic lipopeptides, Pam2CSK4 and Pam3CSK4 (EMC, Tübingen, Germany); or 5) a mixture of the LS-1 *S*. *aureus* strain with either Lpl1(+sp) or LS-1 in PBS; or 6) heat-treated Lpl1(+sp) or Pam3CSK4 or unheated Lpl1(+sp) or Pam3CSK4. For experiments with heat-treated purified compounds, Lpl1(+sp) and Pam3CSK4 were heat-treated at 95°C for 45 min. The severity of the clinical arthritis was judged by measuring the difference between the diameters of the knee joints with a caliper every 2–3 days.

### Synovium collection and flow cytometry

Knee synovial tissue was collected from C57Bl/6 wild-type and TLR2^-/-^ mice that received i.a. injection of Lpl1(+sp) (5 μg/knee) or PBS and was placed in RPMI medium (Fisher Scientific). The tissue was resuspended in medium with DNase I (Sigma-Aldrich) and type IV collagenase (Fisher Scientific) and was incubated for 1 hour at 37°C. A single-cell suspension was obtained after the tissue was homogenized and passed through a 35 μm cell strainer (Becton Dickinson). Synovial cells were then analyzed using the following antibodies: V450-conjugated anti-CD11b (Becton Dickinson), allophycocyanin (APC)-conjugated anti-F4/80 (BioLegend), PerCP-Cy5.5-conjugated anti–Gr-1 (BioLegend), fluorescein isothiocyanate (FITC)-conjugated anti-CD3 (BioLegend), and PerCP-conjugated anti-CD19 (BioLegend). Cells were analyzed using a FACSverse flow cytometer (Becton Dickinson). FlowJo version 10.1 software (Tree Star, Ashland, USA) was used to analyze the data.

### *In vivo* cell depletion procedures

Clodronate liposomes (Liposoma BV, Netherlands) are known to function as selective eliminators of macrophages [44]. NMRI mice were i.a. injected in the knee joints with a volume of 20 μl of clodronate liposomes or PBS control liposomes (Liposoma BV, Netherlands) 1 day prior to challenge with Lpl1 or coinjections of SA113*Δlgt* mutant with either Lpl1 or PBS. The mice were also treated intravenously with 200 μl of clodronate liposomes or PBS control liposomes the day before the challenge and on days +1, +3, and +5 after the challenge.

Anti-Ly6G (clone 1A8; BioXCell), a specific monoclonal antibody (mAb), is known to selectively deplete murine blood neutrophils [45]. NMRI mice were intraperitoneally (i.p.) injected with a dose of 400 μg of anti-Ly6G or isotype control (clone 2A3; BioXCell) in 200 μl of PBS/mouse the day before the challenge and on days +1 and, +4 after the challenge with Lpl1 or coinjections of SA113*Δlgt* mutant with either Lpl1 or PBS.

CD4 and CD8 T-cells were depleted simultaneously using rat anti-mouse CD4 mAb (clone GK1.5; BioXCell) and rat anti-mouse CD8α (clone 2.43; BioXCell) mAb; a rat IgG2b isotype control (clone LTF-2; BioXCell) served as a control. NMRI mice were i.p. injected with a dose of 400 μg of each antibody in 200 μl of PBS/mouse the day before and the day after challenge with Lpl1.

The efficacy of cell depletion was verified by flow cytometry. The depletion was carried out as described 1 day prior to blood collection. Mouse blood was collected into heparin tubes, erythrocytes were depleted with lysis buffer (0.16 M NH_4_Cl, 0.13 mM EDTA, and 12 mM NaHCO_3_), and samples were washed. The single-cell suspensions were then adjusted in FACS buffer to a density of 2x10^6^ cells/mL and analyzed using the following antibodies: V450-conjugated anti-CD11b, PE-Cy7-conjugated anti-Ly6G, Per-CP-conjugated anti-CD4 and PE-conjugated anti-CD8 (all from Becton Dickinson) APC-conjugated anti-F4/80 (BioLegend). The representative flow cytometry plots are presented in S7 Fig. For cell depletion protocols, 85.6% of monocytes (CD11b+, Ly6G- and F4/80+ white blood cells), 99.6% of neutrophils (CD11b+, Ly6G+, F4/80-), 97.3% of CD11b- CD4+ T cells and 90% of CD11b- CD8+ T cells were depleted (S7 Fig).

### Treatment with TNFα inhibitor, IL-1 receptor antagonist, and CTLA4-Ig in mice with Lpp-induced arthritis

Etanercept (Enbrel; Wyeth Europa), a soluble TNF receptor, was used for the anti-TNF treatment because it fully inhibits the biological function of murine TNF [8, 11]. Abatacept (Orencia; Bristol-Myers Squibb), a fusion protein of CTLA4-Ig, was used to modulate the costimulation of T-cells in mice [11, 46]. Anakinra (Kineret; Amgen), an IL-1 receptor antagonist, was used to block murine IL-1 [10, 47]. Etanercept (0.2 mg/mouse in 0.1 mL of PBS) or abatacept (0.5 mg/mouse in 0.1 mL of PBS) was given subcutaneously (s.c.) the day before and the day after challenge with Lpl1. Anakinra (1 mg/mouse in 0.1 mL of PBS) was given s.c. daily, starting from day -1 until day +2 after the challenge. PBS served as a control.

### Treatment with TNFα inhibitor, iNOS inhibitor and anti-CXCR2 in mice coinjected with *S*. *aureus* SA113*Δlgt* and Lpl1

A potent selective inhibitor of iNOS, 1400W, was used to block murine iNOS [48]. Etanercept (0.1 mg/mouse in 0.1 mL of PBS) or 1400W (0.25 mg/mouse in 0.1 mL of PBS) was s.c. injected into NMRI mice twice per day, starting on day -1 until day +2 after challenge with coinjections of SA113*Δlgt* mutant with either Lpl1 or PBS. PBS treatment served as a control for both groups.

Monoclonal anti-mouse CXCR2 antibody (clone 242216; R&D Systems) was used to block murine CXCR2 [49]. NMRI mice were i.p. injected with either anti-CXCR2 or an isotype control antibody (clone 2A3; BioXCell) (75–95 μg/mouse in 0.2 mL of PBS) the day before challenge with coinjections of SA113*Δlgt* mutant with either Lpl1 or PBS.

### Homogenate preparation and bacteriological examination

Knee joints were homogenized with an Ultra Turrax T25 homogenizer (IKA, Staufen, Germany). Then, the homogenate was diluted in PBS, spread on horse blood agar plates, and incubated for 24 hours at 37°C. Viable counts of bacteria were performed and quantified as CFUs.

### Assessment of Lpl1 and Pam3CSK4 influence on *S*. *aureus* growth

SA113*Δlgt* mutant bacteria (10^3^ CFU/mL) were incubated with 25 μg/mL of Lpl1, 100 μg/mL of Pam3CSK4, or PBS control in tryptic soy broth (TSB) medium. At specific time intervals (1, 3, 6, and 24 hours), samples of the bacterial mixtures (100 μl) were spread on horse blood agar plates. After incubation for 18 hours at 37°C, colonies were counted. The effect of exogenous Lpl1 and Pam3CSK4 on *S*. *aureus* growth was evaluated by comparing the number of CFUs in the PBS control and the Lpl1- or Pam3CSK4-treated staphylococcal cultures at the different time points.

### Whole blood killing assay

Blood samples from healthy NMRI mice (n = 4) were collected into heparin-containing tubes. SA113 or SA113*Δlgt* mutant bacterial suspensions were prepared and added into the mouse blood to a final concentration of approximately 1x10^3^ CFU/mL. The incubated mixtures were shaken at 300 rpm for 2 hours at 37°C. To determine bacterial viability in blood, aliquots were withdrawn after 0, 30, 60 and 120 minutes of incubation, and samples were plated onto horse blood agar plates. Bacterial survival was evaluated as a percentage of number of CFUs at different time points compared with the number of bacteria initially added to the whole blood.

### *In vitro* splenocyte and peritoneal macrophage stimulation

Splenocytes from healthy NMRI mice and peritoneal macrophages from C57Bl/6 and TLR2^-/-^ mice were prepared under sterile conditions. To prepare the splenocyte culture, mouse spleens were aseptically removed and passed through a nylon mesh.

To collect the peritoneal macrophages, peritoneal lavage was performed using 10 mL of ice cold PBS. Erythrocytes were depleted in both cultures by lysis in 0.83% ammonium chloride, and the remaining cells were washed in PBS. The single-cell suspensions were then adjusted in Iscove’s complete medium (10% fetal calf serum, 2 mM L-glutamine, 5x10^-5^ M mercaptoethanol and 50 μg/mL gentamicin) to a density of 1.5x10^6^ cells/mL for splenocytes and 5x10^5^ cells/mL for macrophages.

For the proliferation assay, the splenocytes were stimulated with purified Lpl1(+sp) or Lpl1(-sp) (20–200 ng/mL), Pam2CSK4 or Pam3CSK4 (40 ng/mL), and TSST-1 (100 ng/mL) or Iscove's medium (negative control). A total of 1 μl of Ci [^3^H]thymidine (Amersham, Bucks, UK) was added for incorporation 12 hours before the cells were harvested, and the proliferative response was read with a micro-β counter. For cytokine/chemokine analysis, the macrophages were stimulated with purified Lpl1(+sp) or Lpl1(-sp) (0.02–0.2 μg/mL), Pam3CSK4 (2–20 ng/mL), LPS (1 μg/mL), or Iscove's medium for 4 hours. The supernatants were saved for later analysis.

### Lactate dehydrogenase (LDH) cytotoxicity assay

Splenocytes (5x10^6^ cells/mL) from healthy NMRI mice (n = 4) were incubated with either 5x10^6^ CFU/mL (multiplicity of infection [MOI] = 1) or 25x10^6^ CFU/mL (MOI = 5) of SA113 or SA113*Δlgt* mutant bacteria in Iscove’s complete medium for 6 hours at 37°C. Aliquots were collected at 0.5, 1, 3, and 6 hours of incubation. LDH was measured in the supernatants with a cytotoxicity detection kit (Roche Diagnostics GmbH, Mannheim, Germany), according to the manufacturer’s directions. Absorbance was measured at 490 nm, and the results show the percentage of maximal LDH release in relation to positive control (splenocytes treated with Triton X-100).

### Phagocytosis analysis

Peritoneal macrophages from NMRI mice were adjusted in Kreb’s Ringers glucose (KRG) (1x10^6^ cells/mL) and stimulated with purified Lpl1(+sp) (0.2 μg/mL) or PBS at 37°C for 1 hour. To study whether Lpl1 impacts phagocytosis, green fluorescent protein (GFP)-expressing *S*. *aureus* in KRG was incubated with or without 20% mouse serum at 37°C for 30 min, mixed with the stimulated peritoneal macrophages, incubated at 37°C for 20 min and analyzed as previously described [50].

### Measurement of cytokine and chemokine levels

The levels of IL-6, MIP-2, KC and TNFα in the supernatants from the knee joint homogenates and the levels of KC, MIP-2, MCP-1 and TNFα in the supernatants from peritoneal macrophage or splenocyte stimulation were quantified using DuoSet ELISA Kits (R&D Systems, Abingdon, UK).

### Microcomputed tomography (μCT)

The knee joints were fixed in 4% formaldehyde for 3 days and then transferred to PBS. Imaging of the knee joints was performed *ex vivo* with a Skyscan1176 **μ**CT scanner (Bruker, Antwerp, Belgium) to detect bone destruction after the studies were completed. The **μ**CT settings were adjusted to a voxel size of 18 **μ**m, an aluminum filter of 0.2 mm, and an exposure time of 800 ms, and the scans were conducted at 45 kV/555 **μ**A. The X-ray projections were obtained at 0.42° intervals with a scanning angular rotation of 180°. The projection images were reconstructed into three-dimensional images using NRECON software (version 1.6.9.8; Bruker) and analyzed with CT-Analyzer (version 2.7.0; Bruker). After reconstruction, experienced observers (M.M. and T.J.) evaluated the extent of bone and cartilage destruction in a blinded manner on a grading scale from 0–3 as previously described [11].

### Histopathological examination

After the **μ**CT scanning, the joints were decalcified, embedded in paraffin and sectioned with a microtome. Tissue sections were thereafter stained with hematoxylin and eosin. All slides were coded and assessed under a microscope in a blinded manner by two observers (T.J. and M.M.). The extent of synovitis and cartilage-bone destruction was judged as previously described [12].

### Statistical analysis

Statistical significance was assessed using the Mann-Whitney U test and Fisher's exact test, as appropriate. The results are reported as the mean ± standard error of the mean (SEM) unless indicated otherwise. A *p* value <0.05 was considered statistically significant. Calculations were performed using GraphPad Prism version 7.0b software for Macintosh (GraphPad software, La Jolla, CA, USA).

## Supporting information

S1 Fig Heat-denaturation of staphylococcal lipoproteins and lipopeptide has no impact on their arthritogenic properties. Purified Lpl1(+sp) or Pam3CSK4 were heat-treated at 95°C for 45 min. 20 μl of heat-treated and unheated Lpl1(+sp) (5 μg/knee) or Pam3CSK4 (5 μg/knee) were i.a. injected into NMRI mice knee joints (n = 2-3/group). The severity of the clinical arthritis induced by (**A**) Lpl1 or (**B**) Pam3CSK4 was assessed by measuring the difference between the diameters of the knee joints up to 3 days. Statistical evaluations were performed using the Mann–Whitney U test, with data expressed as the mean ± standard error of the mean.
(TIF)

S2 Fig *S*. *aureus* SA113*Δlgt* mutant has similar cytotoxicity as SA113 strain towards mouse splenocytes. Splenocytes (5x10^6^ cells/mL) from healthy NMRI mice (n = 4) were incubated with either (**A**) 5x10^6^ CFU/mL (multiplicity of infection [MOI] = 1) or (**B**) 25x10^6^ CFU/mL (MOI = 5) of SA113 or SA113*Δlgt* mutant bacteria in Iscove’s complete medium for 6 hours at 37°C. Aliquots were collected at 0.5, 1, 3, and 6 hours of incubation for analyses of LDH release, and the results show the percentage of maximal LDH release in relation to positive control (splenocytes treated with Triton X-100). Statistical evaluations were performed using the Mann–Whitney U test, with data expressed as the mean ± standard error of the mean.
(TIF)

S3 Fig Neither *S*. *aureus* lipoproteins nor lipopeptides exert direct bactericidal effect. SA113*Δlgt* mutant bacteria (10^3^ CFU/mL) were incubated with 25 μg/mL of Lpl1, 100 μg/mL of Pam3CSK4, or PBS control in tryptic soy broth (TSB) medium. At specific time intervals (1, 3, 6, and 24 hours), the effect of (**A**) exogenous Lpl1 and (**B**) Pam3CSK4 on *S*. *aureus* growth was evaluated by comparing the number of CFUs between the PBS control and the Lpl1- or Pam3CSK4-treated staphylococcal cultures. Statistical evaluations were performed using the Mann–Whitney U test, with data expressed as the mean ± standard error of the mean.
(TIF)

S4 Fig The phagocytic capacity of mouse peritoneal macrophages is not influenced by staphylococcal lipoproteins. Peritoneal leukocytes obtained by peritoneal lavage from NMRI mice were stimulated with purified staphylococcal lipoprotein, denoted as Lpl1(+Lpl1) (0.2 μg/mL) or PBS (-Lpl1) at 37°C for 1 hour and incubated with GFP-expressing *S*. *aureus* (multiplicity of infection [MOI] = 5) with or without serum opsonization. The IDEAS software internalization wizard was used to determine the interaction of the GFP-positive bacteria with phagocytes (not associated, surface bound, or internalized). **(A)** Percentages of peritoneal macrophages engulfing GFP-positive *S*. *aureus* with and without opsonization. **(B)** Representative image of peritoneal macrophages associated with GFP-expressing *S*. *aureus* (MOI = 5) analyzed by flow cytometry imaging.
(TIF)

S5 Fig *S*. *aureus* SA113*Δlgt* mutant has similar survival rate as SA113 strain in whole blood. Whole blood samples from healthy NMRI mice (n = 4) were incubated with SA113 or SA113*Δlgt* mutant bacteria in a final concentration of approximately 1x10^3^ CFU/mL. To determine bacterial viability in blood, aliquots were withdrawn after 0, 30, 60 and 120 minutes of incubation. Bacterial survival was evaluated as a percentage of number of CFUs at different time points compared with the number of bacteria initially added to the whole blood. Statistical evaluations were performed using the Mann–Whitney U test, with data expressed as the mean ± standard error of the mean.
(TIF)

S6 Fig Lipid moiety of lipoproteins induces TNFα production in peritoneal macrophages and splenocytes from mice. The levels of TNFα in the supernatants collected from C57BL/6 wildtype (WT) and TLR-2 deficient (TLR2-/-) mouse peritoneal macrophage cell cultures (5x10^5^ cells/mL) **(A)** and splenocyte cultures (1x10^6^ cells/mL) **(B)** after stimulation with Lpl1(+sp) (0.02–0.2 μg/ml); unlipidated Lpl1 protein, denoted as Lpl1(-sp) (0.02–0.2 μg/ml); Pam3CSK4 (2–20 ng/ml); LPS (1 μg/ml); or culture medium for 24 hours. Statistical evaluations were performed using the Mann–Whitney U test, with data expressed as the mean ± standard error of the mean.
(TIF)

S7 Fig The different cell types were effectively depleted as confirmed by flow cytometry analysis. NMRI mice were treated with 1) clodronate liposomes to deplete monocytes/macrophages; 2) anti-mouse Ly6G monoclonal antibody (mAb) to deplete neutrophils; and 3) anti-mouse CD4 mAb and anti- mouse CD8α mAb to deplete T cells. The blood was collected one day after treatment. Representative images of fluorescence-activated cell sorting (FACS) analysis demonstrating the efficacy of cell depletion for (**A**) monocytes/macrophages (CD11b+F4/80+Ly6G-), **(B)** neutrophils (CD11b+Ly6G+F4/80-), and (**C**) T cells (CD11b-CD4+CD8+).
(TIF)
