# Supplementary figures and images for: The YIN and YANG of lipoproteins in developing and preventing infectious arthritis by Staphylococcus aureus
Source: PLoS Pathog. 2019 Jun 21;15(6):e1007877. doi: 10.1371/journal.ppat.1007877 (PMC6608979; doi:10.1371/journal.ppat.1007877)

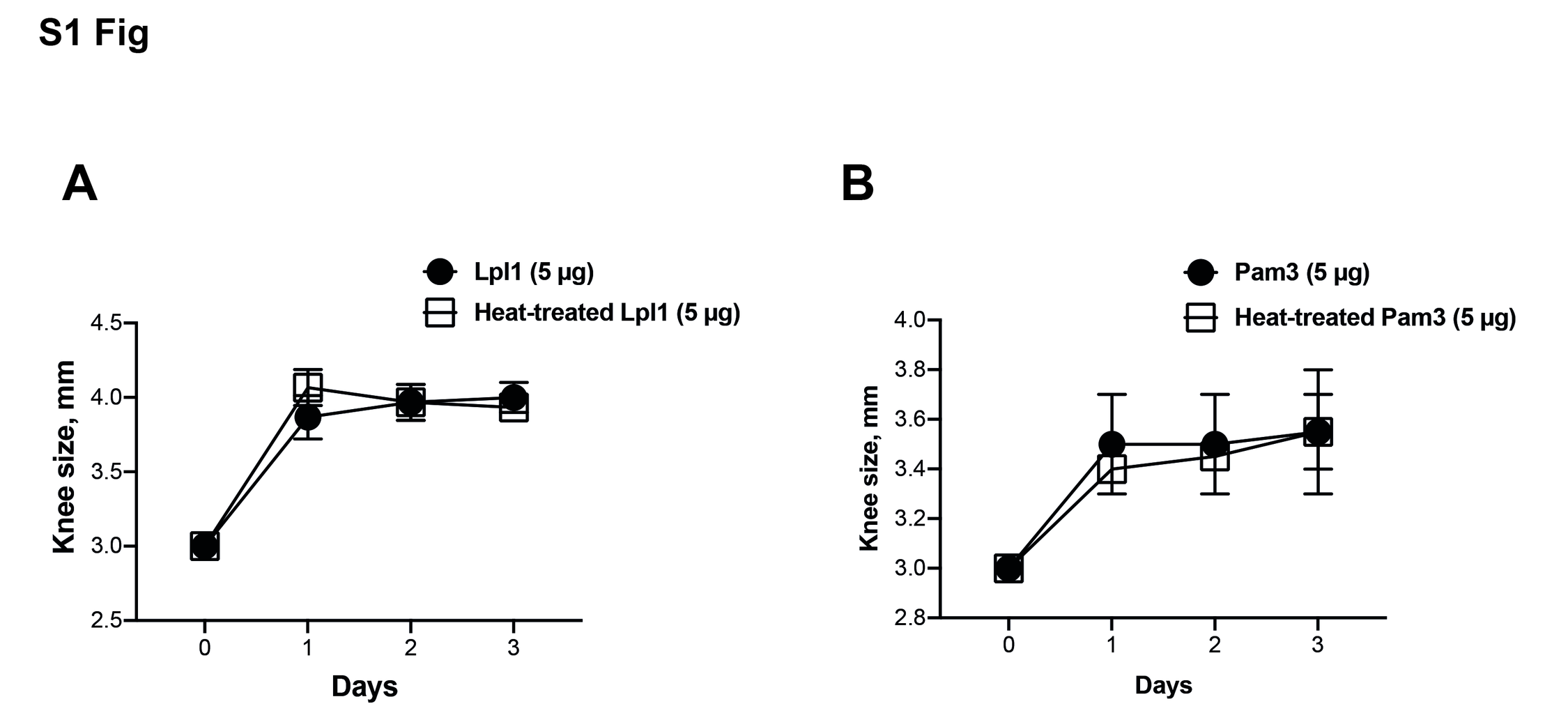

Supplement: S1 Fig — Purified Lpl1(+sp) or Pam3CSK4 were heat-treated at 95°C for 45 min. 20 μl of heat-treated and unheated Lpl1(+sp) (5 μg/knee) or Pam3CSK4 (5 μg/knee) were i.a. injected into NMRI mice knee joints (n = 2-3/group). The severity of the clinical arthritis induced by (A) Lpl1 or (B) Pam3CSK4 was assessed by measuring the difference between the diameters of the knee joints up to 3 days. Statistical evaluations were performed using the Mann–Whitney U test, with data expressed as the mean ± standard error of the mean. (TIF) [file ppat.1007877.s001.tif]

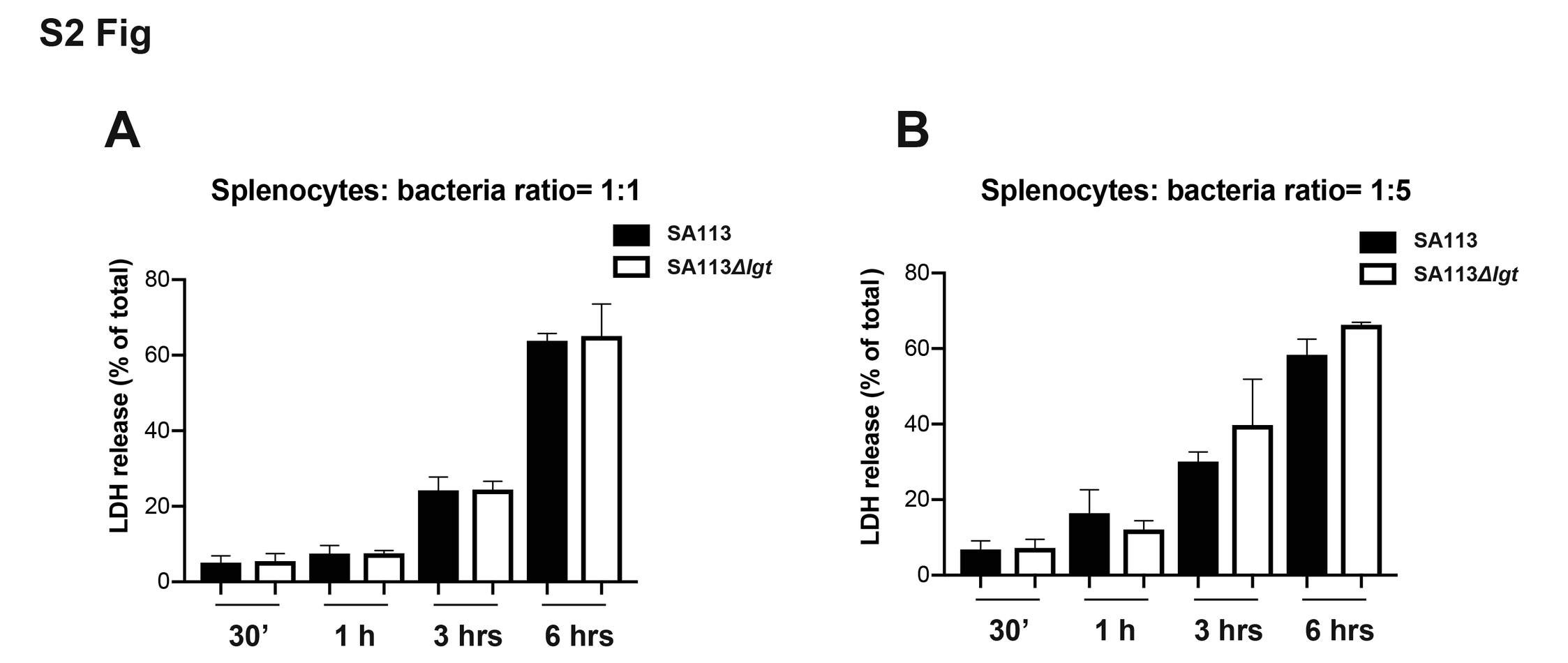

Supplement: S2 Fig — Splenocytes (5x10^6^ cells/mL) from healthy NMRI mice (n = 4) were incubated with either (A) 5x10^6^ CFU/mL (multiplicity of infection [MOI] = 1) or (B) 25x106 CFU/mL (MOI = 5) of SA113 or SA113Δlgt mutant bacteria in Iscove’s complete medium for 6 hours at 37°C. Aliquots were collected at 0.5, 1, 3, and 6 hours of incubation for analyses of LDH release, and the results show the percentage of maximal LDH release in relation to positive control (splenocytes treated with Triton X-100). Statistical evaluations were performed using the Mann–Whitney U test, with data expressed as the mean ± standard error of the mean. (TIF) [file ppat.1007877.s002.tif]

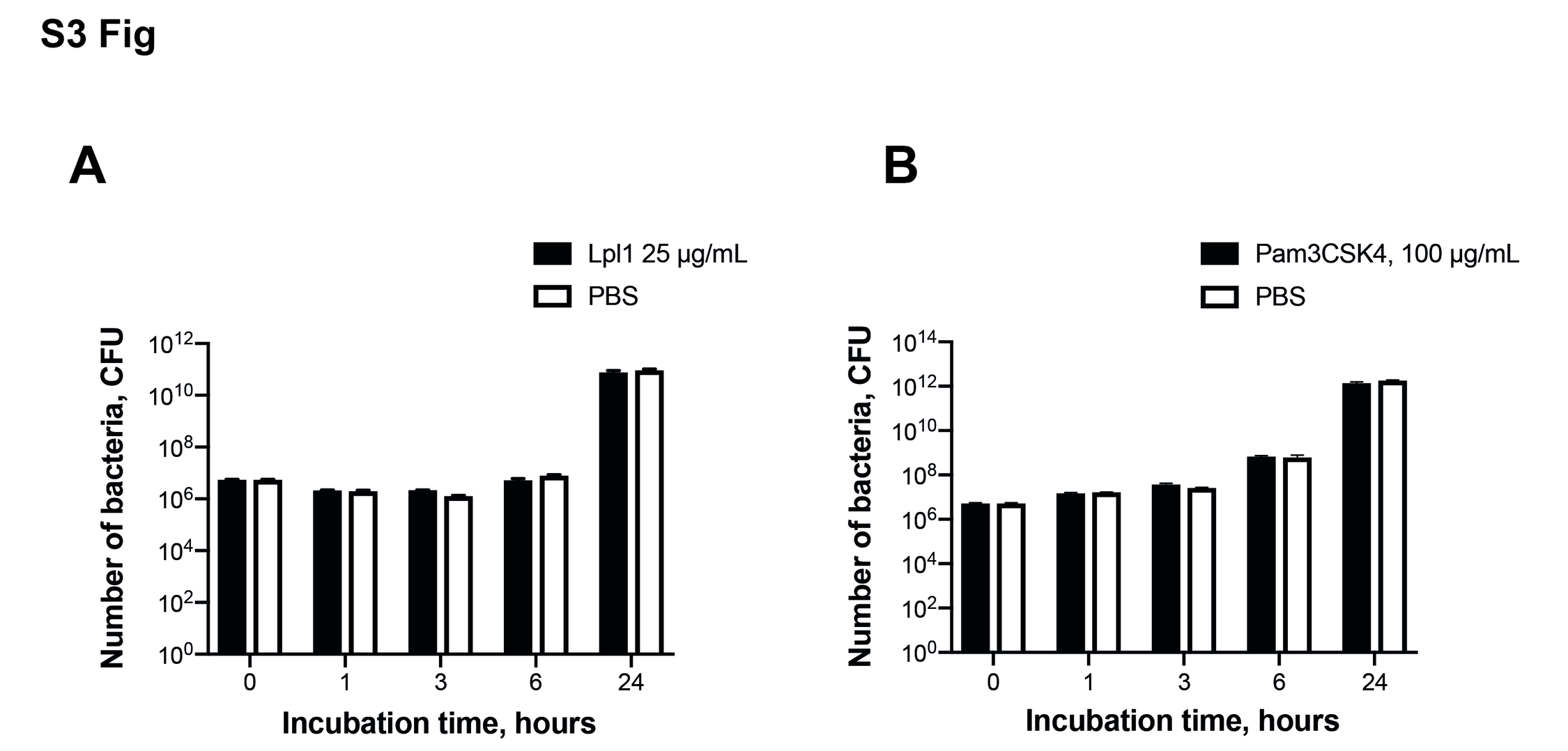

Supplement: S3 Fig — SA113Δlgt mutant bacteria (103 CFU/mL) were incubated with 25 μg/mL of Lpl1, 100 μg/mL of Pam3CSK4, or PBS control in tryptic soy broth (TSB) medium. At specific time intervals (1, 3, 6, and 24 hours), the effect of (A) exogenous Lpl1 and (B) Pam3CSK4 on S. aureus growth was evaluated by comparing the number of CFUs between the PBS control and the Lpl1- or Pam3CSK4-treated staphylococcal cultures. Statistical evaluations were performed using the Mann–Whitney U test, with data expressed as the mean ± standard error of the mean. (TIF) [file ppat.1007877.s003.tif]

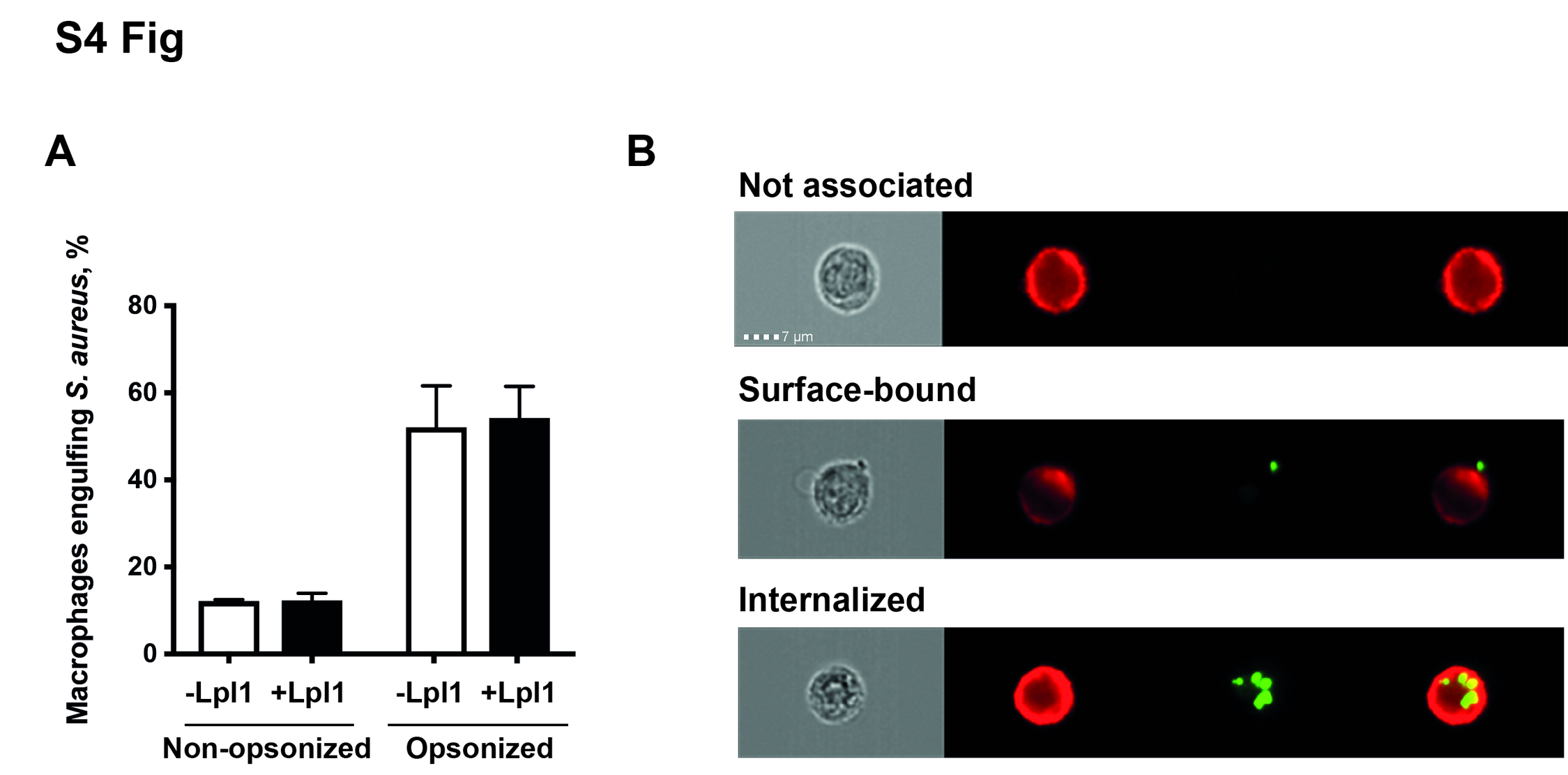

Supplement: S4 Fig — Peritoneal leukocytes obtained by peritoneal lavage from NMRI mice were stimulated with purified staphylococcal lipoprotein, denoted as Lpl1(+Lpl1) (0.2 μg/mL) or PBS (-Lpl1) at 37°C for 1 hour and incubated with GFP-expressing S. aureus (multiplicity of infection [MOI] = 5) with or without serum opsonization. The IDEAS software internalization wizard was used to determine the interaction of the GFP-positive bacteria with phagocytes (not associated, surface bound, or internalized). (A) Percentages of peritoneal macrophages engulfing GFP-positive S. aureus with and without opsonization. (B) Representative image of peritoneal macrophages associated with GFP-expressing S. aureus (MOI = 5) analyzed by flow cytometry imaging. (TIF) [file ppat.1007877.s004.tif]

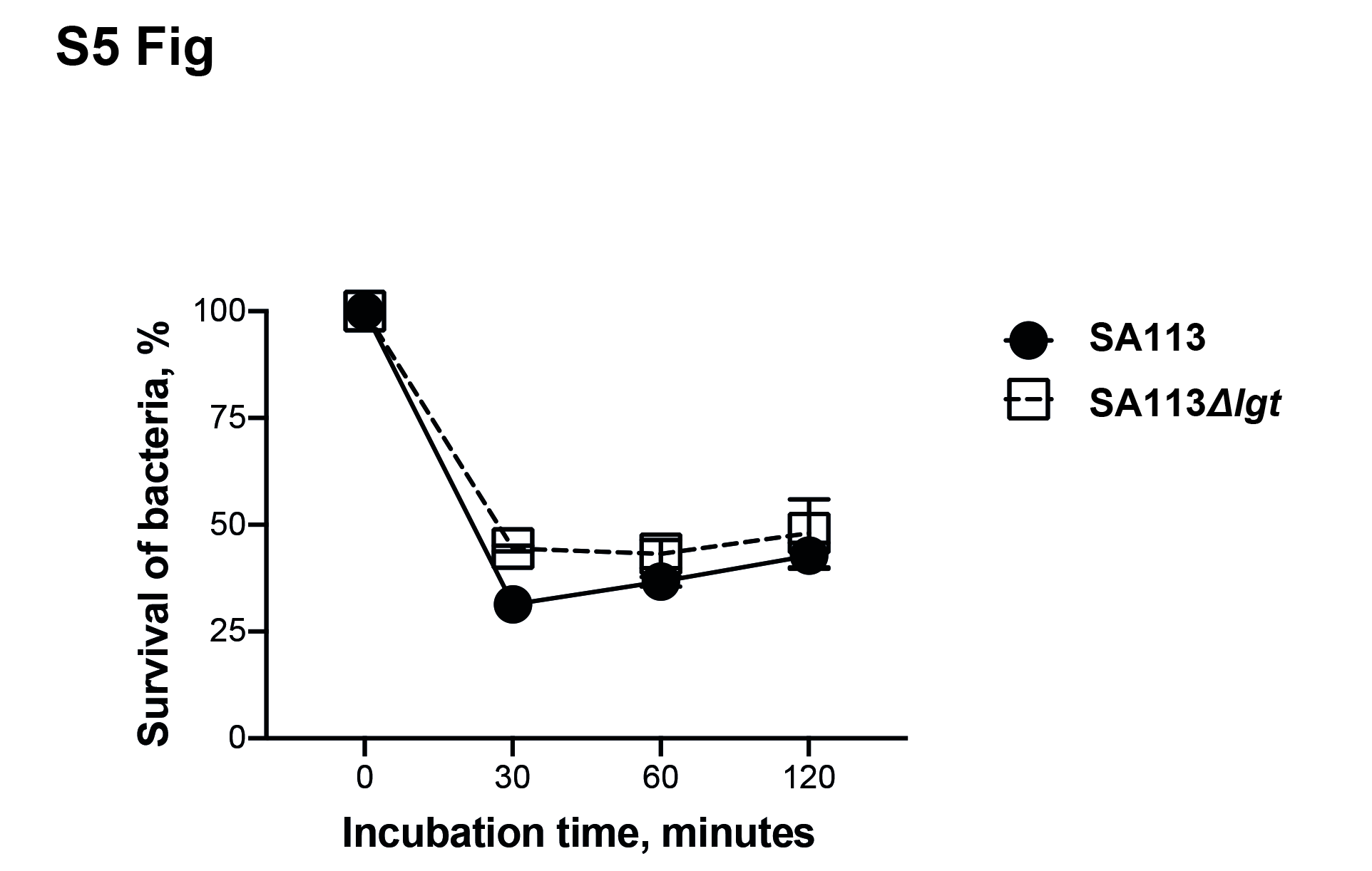

Supplement: S5 Fig — Whole blood samples from healthy NMRI mice (n = 4) were incubated with SA113 or SA113Δlgt mutant bacteria in a final concentration of approximately 1x10^3^ CFU/mL. To determine bacterial viability in blood, aliquots were withdrawn after 0, 30, 60 and 120 minutes of incubation. Bacterial survival was evaluated as a percentage of number of CFUs at different time points compared with the number of bacteria initially added to the whole blood. Statistical evaluations were performed using the Mann–Whitney U test, with data expressed as the mean ± standard error of the mean. (TIF) [file ppat.1007877.s005.tif]

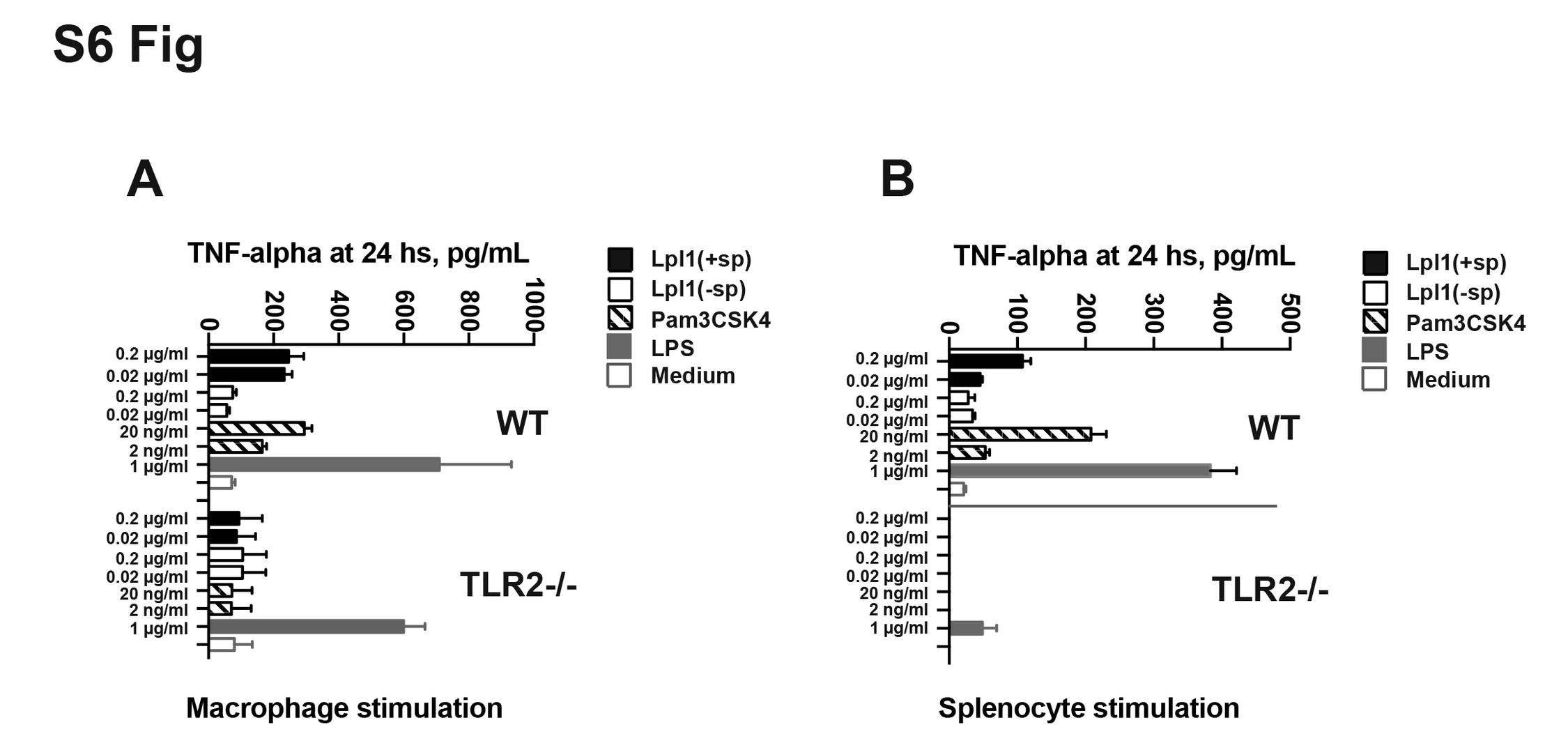

Supplement: S6 Fig — The levels of TNFα in the supernatants collected from C57BL/6 wildtype (WT) and TLR-2 deficient (TLR2-/-) mouse peritoneal macrophage cell cultures (5x10^5^ cells/mL) (A) and splenocyte cultures (1x10^6^ cells/mL) (B) after stimulation with Lpl1(+sp) (0.02–0.2 μg/ml); unlipidated Lpl1 protein, denoted as Lpl1(-sp) (0.02–0.2 μg/ml); Pam3CSK4 (2–20 ng/ml); LPS (1 μg/ml); or culture medium for 24 hours. Statistical evaluations were performed using the Mann–Whitney U test, with data expressed as the mean ± standard error of the mean. (TIF) [file ppat.1007877.s006.tif]

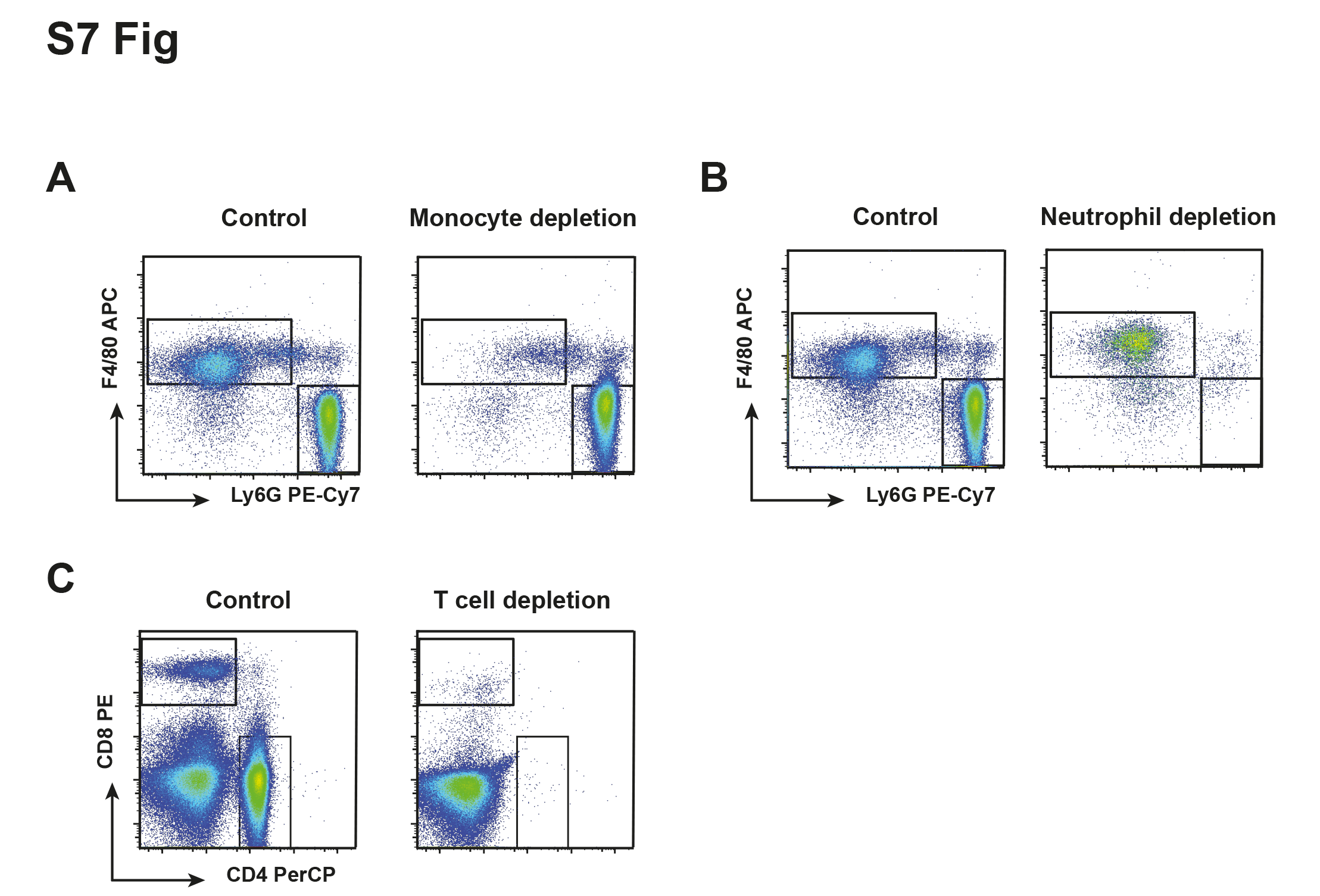

Supplement: S7 Fig — NMRI mice were treated with 1) clodronate liposomes to deplete monocytes/macrophages; 2) anti-mouse Ly6G monoclonal antibody (mAb) to deplete neutrophils; and 3) anti-mouse CD4 mAb and anti- mouse CD8α mAb to deplete T cells. The blood was collected one day after treatment. Representative images of fluorescence-activated cell sorting (FACS) analysis demonstrating the efficacy of cell depletion for (A) monocytes/macrophages (CD11b+F4/80+Ly6G-), (B) neutrophils (CD11b+Ly6G+F4/80-), and (C) T cells (CD11b-CD4+CD8+). (TIF) [file ppat.1007877.s007.tif]
